# Composition and global distribution of the mosquito virome - A comprehensive database of insect-specific viruses

**DOI:** 10.1016/j.onehlt.2023.100490

**Published:** 2023-01-20

**Authors:** Jurgen P. Moonen, Michelle Schinkel, Tom van der Most, Pascal Miesen, Ronald P. van Rij

**Affiliations:** Department of Medical Microbiology, Radboud Institute for Molecular Life Sciences, Radboud University Medical Center, P.O. Box 9101, 6500 HB Nijmegen, the Netherlands

**Keywords:** Virome, Insect-specific viruses, Mosquito, Aedes, Culex, Anopheles, Metagenomics

## Abstract

Mosquitoes are vectors for emerging and re-emerging infectious viral diseases of humans, livestock and other animals. In addition to these arthropod-borne (arbo)viruses, mosquitoes are host to an array of insect-specific viruses, collectively referred to as the mosquito virome. Mapping the mosquito virome and understanding if and how its composition modulates arbovirus transmission is critical to understand arboviral disease emergence and outbreak dynamics. In recent years, next-generation sequencing as well as PCR and culture-based methods have been extensively used to identify mosquito-associated viruses, providing insights into virus ecology and evolution. Until now, the large amount of mosquito virome data, specifically those acquired by metagenomic sequencing, has not been comprehensively integrated. We have constructed a searchable database of insect-specific viruses associated with vector mosquitoes from 175 studies, published between October 2000 and February 2022. We identify the most frequently detected and widespread viruses of the *Culex, Aedes* and *Anopheles* mosquito genera and report their global distribution. In addition, we highlight the challenges of extracting and integrating published virome data and we propose that a standardized reporting format will facilitate data interpretation and re-use by other scientists. We expect our comprehensive database, summarizing mosquito virome data collected over 20 years, to be a useful resource for future studies.

## 1 Introduction

Hematophagous mosquitoes are vectors for the transmission of arthropod-borne viruses (arboviruses) to humans, livestock and wild animals. Particularly, mosquitoes of the genera *Aedes* and *Culex* transmit epidemic arboviruses, including dengue virus, yellow fever virus, Zika virus and West Nile virus [[1], [2], [3]]. Mosquitoes of the genus *Anopheles* are the main vector for O'nyong-nyong virus as well as for malaria parasites [4]. Besides arboviruses, which have a dual host range alternating between vertebrates and arthropods, mosquitoes carry viruses with an insect-restricted host range (insect-specific viruses, ISVs), as well as viruses that infect microbes such as the bacteria and fungi that colonize the mosquito host [5,6]. These mosquito-associated viruses are collectively referred to as the mosquito virome.

Virome studies of mosquitoes, and invertebrates in general, have shed light on the vast diversity of viruses on earth [7,8]. In recent years, next-generation sequencing, PCR-based detection, and virus culture approaches have been extensively used to map the virome across mosquito genera, ecological environments, and geographical locations. These studies expanded the host range of virus families to include arthropods (e.g., in the *Totiviridae* and *Partitiviridae* families [[9], [10], [11]]), introduced new clades within existing viral families or orders (e.g., *Artivirus* in the *Totiviridae* [9] and *Goukovirus*, *Herbevirus*, *Jonvirus* and *Feravirus* in the *Bunyavirales* [[12], [13], [14]]), and necessitated the creation of novel viral families and genera (e.g., *Mesoniviridae* and *Negevirus* [[15], [16], [17], [18]]). Additionally, fundamental insights into virus evolution may be obtained from these studies, as exemplified by the discovery of Nam Dinh virus (or alphamesonivirus 1) in mosquitoes, which led to the establishment of a new family *Mesoniviridae* in the order *Nidovirales*, containing viruses with a genome size intermediate between the small-sized *Arteriviridae* and the large-sized *Coronaviridae* and *Roniviridae* [16].

The mosquito virome has raised significant interest because of its potential impact on the transmission of arboviruses or malarial parasites [[19], [20], [21], [22], [23], [24]]. Correlating spatiotemporal virome data with vector-borne disease incidence may provide insights into the impact of ISVs on pathogen transmission. Moreover, for many ISVs, the host range and the potential to cross the species barrier and infect other (vertebrate or invertebrate) animals remains to be established. Virus infection may impact mosquito physiology and development, which is almost completely uncharacterized thus far but may, directly or indirectly, affect vectorial capacity. Thus, for a One Health perspective on arbovirus transmission, a systematic overview of the prevalence of mosquito-specific viruses is essential. Such an overview may also inform biotechnological applications of ISVs, such as the development of novel vaccine platforms, or their use as biological agents to prevent arbovirus transmission by mosquitoes [22,[25], [26], [27]].

Arboviruses are mainly transmitted horizontally between mosquito and vertebrate hosts. In contrast, during adverse conditions such as cold winters or drought, it is hypothesized that arboviruses are vertically transmitted, even if it may be relatively inefficient [28,29]. ISVs are often assumed to be transmitted vertically from parent to offspring, but direct experimental support for this transmission mode is scarce and both vertical and horizontal transmission routes have been proposed [30,31]. Virome studies could be used to deduce transmission modes. For example, frequent recovery of an ISV from early life stages such as eggs or larvae could be indicative of vertical transmission, whereas recovery of the same virus from different mosquito species would suggest a horizontal transmission mode via the environment, such as shared food sources.

It is likely that the virome differs between mosquito species, between populations of the same species of mosquitoes, and between individual mosquitoes within populations, which may depend on the transmission mode as well as on viral and host genetics, mosquito ecology, and environmental and climatic conditions. Yet, some ISVs may be present in mosquito populations across the globe or have a broad mosquito host range. For those viruses, it will be particularly relevant to determine their impact on mosquito physiology, development and pathogen transmission.

The large amount of mosquito virome information has thus far been integrated at different levels of analysis. Some studies compared their acquired metagenomic data with sequencing data from other studies [[32], [33], [34], [35]] and several reviews have collated lists of (insect-specific) viruses detected in mosquitoes [5,25,36]. However, an exhaustive analysis of mosquito-associated viruses, including their location and associated mosquito hosts, is lacking. In this study, we performed a comprehensive review of 175 mosquito virome studies, published between October 2000 and February 2022, to construct a searchable database of mosquito-associated viruses. We present the most widespread and frequently detected insect-specific viruses within the *Culex*, *Aedes* and *Anopheles* mosquito genera and highlight viruses with a particularly broad mosquito host range. We expect our database to be a useful resource for further study of insect-specific viruses.

## 2 Methods

### 2.1 Search strategy

A PubMed search was performed on January 26, 2022, using a combination of title/abstract (Tiab) search terms and Medical Subject Headings (MeSH) terms. The search strategy combined the following terms for (insect-specific) virus discovery with terms for mosquito research:

(“Virome”[MeSH Terms] OR “Metagenomics”[MeSH Terms] OR “Insect Viruses”[MeSH Terms] OR “Metatranscriptomic*”[Title/Abstract] OR “Meta transcriptomic*”[Title/Abstract] OR “Metagenom*”[Title/Abstract] OR “Insect Specific Virus*”[Title/Abstract] OR “ISV”[Title/Abstract] OR “Virus Discovery”[Title/Abstract] OR “Virom*”[Title/Abstract] OR “Insect Specific Flavivirus*”[Title/Abstract] OR “Insect Specific Alphavirus*”[Title/Abstract] **AND** (“Culicidae”[MeSH Terms] OR “Culicid*”[Title/Abstract] OR “Aedes”[Title/Abstract] OR “Anophel*”[Title/Abstract] OR “Culex”[Title/Abstract] OR “Mosquit*”[Title/Abstract]).

The search strategy retrieved 743 articles, which were manually screened. All articles written in English and reporting primary data on virus detection or identification in wild-caught mosquitoes were eligible for the analysis. Articles that only detected arboviruses in mosquitoes were excluded, leading to a final selection of 175 articles (references in Supplementary file 1).

### 2.2 Database assembly

Information on mosquito-associated viruses was extracted from the articles at the level of individual samples, containing either a single mosquito or a pool of mosquitoes, to construct a sample-structured database (Supplementary Table S1). Known arboviruses were not included in the table. Each entry in the database constitutes a virus detected in a mosquito sample. Samples tested negative for viruses were not included in the database. For each virus-positive sample, virus taxonomy at the family level, the mosquito species in which the virus was detected, sampling location, blood-feeding status, method for virus detection, material used for sequencing (RNA, DNA or both), the number of mosquitoes in the sample, and the developmental stage (larva, pupa, adult) was extracted from the articles, if this information could be unambiguously deduced. For consistency, *Culex pipiens* was used for studies reporting *Culex pipiens complex* and *Culex pipiens sensu lato* [37,38]. Likewise, *Ochlerotatus caspius* and *Ochlerotatus scapularis* were denoted as *Aedes caspius* and *Aedes scapularis*, respectively, as both genus names were used in the literature [39]. Virus detection methods were classified into four categories: 1) sequencing, for samples that were directly analyzed by next-generation sequencing, 2) PCR, for samples in which viruses of a particular virus taxon or species were detected by PCR, 3) culture-sequencing, when mosquito homogenate was cultured on mosquito cell lines, after which viruses were detected by next-generation sequencing, and 4) culture-PCR, when mosquito homogenate was cultured on cell lines and virus was detected by PCR using virus taxon or species specific PCR primers.

Information for the database was extracted from the relevant (supplemental) figures or tables as reported. When viruses were not assigned to the species level, but only the closest viral match was reported, these were included in the database. No thresholds were taken into account for the minimal number of reads and genome coverage required for the accurate detection of viruses, with the exception of the study by Hameed et al. [40] (see below). In addition, the authors' assessments were accepted for considering an identified viral sequence novel and giving it a new name. To allow comparison between studies, a column named ‘Virus (clean)’ was defined, in which strain or isolate names from the ‘Virus (reported)’ column were removed, virus abbreviations were written out, and consistent spelling was used.

The NCBI Taxonomy database was used as a reference to define unique viruses, as many ISVs are not yet formally classified by the International Committee on Taxonomy of Viruses (ICTV) and therefore absent from the ICTV Master Species List 2021 [41]. For entries without unambiguous reference to a unique virus, ‘unknown’ was used, except in occasional cases in which the virus name could be deduced from the NCBI taxonomy database using the reported GenBank accession numbers. Virus taxonomy was obtained from the NCBI Taxonomy Database (resourced March through June 2022; [42]) for entries that lack a definition of the virus family in the original article and for viruses with inconsistent taxonomy between articles. In the absence of virus taxonomy at the family level, ‘unknown’ was used.

### 2.3 Database curation

Initial analysis of the contribution of individual publications to the database indicated that one study dominated the dataset, supplying 4169 of the 8378 (50%) total unique virus entries [40] (Supplemental Fig. 1A). This overrepresentation could not be accounted for by the sampling size or sequence depth in this study as only ten pools of mosquitoes were sequenced, which contributed between 121 and 836 virus entries per pool to the database [40]. Instead, for the majority of the reported viruses only a single or few reads were detected and the percentage identity to the viral reference genomes was unreported, providing limited evidence for the presence of these viruses [40]. To prevent a disproportionally large influence of this study on our dataset, a threshold on the minimal number of sequencing reads was applied and only virus entries supported by ≥100 reads were included in the database for this particular study. After this curation, this study supplied 382 of the 4591 (8%) total unique virus entries in our database.

### 2.4 Analyses

Unique virus entries were defined by unique combinations of the columns ‘Study’, ‘Virus family’, ‘Virus (reported)’, ‘Location (Specific)’ and ‘Species’ for the analyses of virus families, or the columns ‘Study’, ‘Virus (clean)’, ‘Location (Specific)’ and ‘Species’ for the analyses at the virus species level. In-house R-scripts were used for data analyses.

## 3 Results

We performed a review of the literature on virus identification in wild-caught mosquitoes. Based on a total of 175 publications, we generated a database consisting of 11,261 rows, each entry representing a virus detected in a specific sample (Supplementary Table S1). The number of virus entries in this database is biased towards studies that acquire a large number of samples from the same location, in particular PCR studies that often test multiple mosquito pools sampled at the same site. To account for these biases, we used a transformed database for our analyses, which only included rows with unique combinations of Study - Virus - Mosquito species - Location. We refer to the rows of this database as unique virus entries (*n* = 4591) and use it as a metric for the abundance of viruses and virus families.

### 3.1 Overview of the literature

**Fig. 1:**
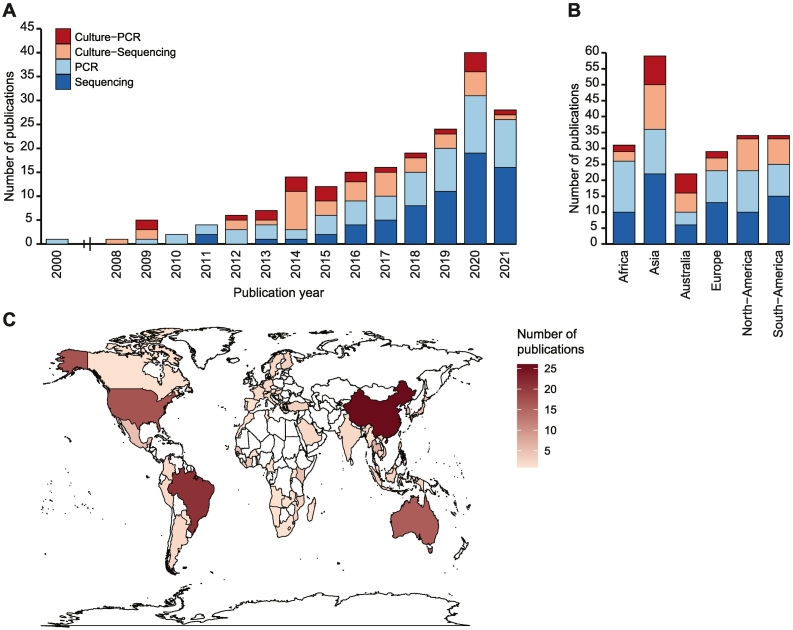
Characteristics of mosquito virome studies. (A-B) Number of mosquito virome publications over time (A), and across continents (B), with fill color indicating the study approach. The sum of the categories within each bar may exceed the actual number of publications as some studies used multiple virus detection methods. (C) Geographic distribution of countries in which mosquitoes were sampled for virome studies.

The number of mosquito virome studies has gradually risen over the years, with two studies published between 2000 and 2008 and a total of 27 studies published in 2021 (Fig. 1A). A slight majority of these studies (*n* = 70) used next-generation sequencing-based approaches to characterize the virome, whereas PCR-based approaches (*n* = 67) were frequently used to specifically detect viruses from genera known to contain arboviruses and/or ISVs, such as flaviviruses, alphaviruses, phleboviruses, orthobunyaviruses, densoviruses and rhabdoviruses (Fig. 1A). In fewer studies, mosquito homogenate was first cultured on mosquito cell lines, often the RNA interference (RNAi)-deficient *Aedes albopictus* C6/36 cell line, followed by PCR (*n* = 21) or next-generation sequencing (*n* = 38) to detect in vitro replicating viruses.

Unique virus entries were not equally distributed across virome studies, with the majority of entries (88.1%) derived from metagenomic sequencing studies and only a small percentage from PCR studies (7.2%) and culturing studies (4.7%). Furthermore, over 50% of the total number unique virus entries were derived from only 10 out of 175 studies (Supplementary Fig. S1A).

**Fig. 2:**
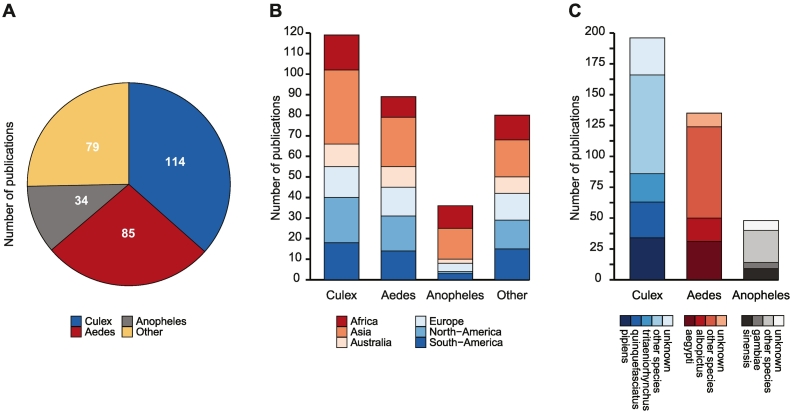
Characteristics of mosquitoes sampled for virome studies. (A) Pie chart indicating the number of publications detecting viruses for each mosquito genus. (B) Number of publications for each mosquito genus with fill color indicating the continent in which mosquitoes were sampled. (C) Number of publications for each mosquito genus, with fill color indicating the most frequently sampled species. Some studies sampled mosquitoes from (A) multiple genera, (B) multiple continents, or (C) multiple species within one mosquito genus, and the sum of publications in each panel therefore exceeds the total number of publications.

Mosquitoes were sampled across the globe (Fig. 2B,C) with a relatively even distribution of PCR and sequencing-based methods (Fig. 1B). Sampling was, however, not uniform across continents, as China, Brazil, and the USA were the main sources of virome information from Asia, South-America and North-America, respectively (Fig. 1C).

### 3.2 Mosquito species sampled for virome studies

The 175 studies collectively detected viruses in 128 different mosquito species from 14 mosquito genera (Supplementary Table S2). Mosquitoes from the *Culex*, *Aedes*, and *Anopheles* genera were most frequently found to harbor viruses, likely because these vector mosquitoes are sampled more often for their importance in pathogen transmission (Fig. 2A). *Culex* mosquitoes contributed most unique virus entries in a total of 114 studies, whereas 85 studies detected viruses in *Aedes* mosquitoes, and 34 studies reported viruses in *Anopheles* mosquitoes (Fig. 2A, Table 1). As few studies detected viruses in other mosquito genera, we did not further analyze the data from those mosquitoes (Supplementary Table S1).

Mosquitoes were collected on every continent except Antarctica, with most extensive sampling in Asia and a clear overrepresentation of *Anopheles* sampling in Asia and Africa (Fig. 2B). Almost half of the studies reporting viruses in *Culex*, detected these viruses in *Culex pipiens*, *Culex quinquefasciatus* and *Culex tritaeniorhynchus*. For *Aedes* and *Anopheles* mosquitoes, most studies detected virus in *Aedes aegypti* and *Aedes albopictus*, and in *Anopheles sinensis* and *Anopheles gambiae*, respectively (Fig. 2C, Supplementary Table S2). Noteworthy, the mosquito species or even the genus was unknown for some virus positive pools, due to studies not defining their species and/or genus [[43], [44], [45]], or due to the use of pooled samples containing multiple species [[46], [47], [48]]. Only 13 studies evaluated the virome in immature developmental stages, such as eggs, larvae, or pupa [32,[49], [50], [51], [52], [53], [54], [55], [56], [57], [58], [59], [60]].

### 3.3 General overview of the mosquito virome

In total, viruses from 102 virus families were reported in all mosquito species combined, although the number of unique virus entries was very low for the majority of these families (Supplementary Fig. S1B). As expected, virus families known to contain ISVs and/or arboviruses were among the top 10 most frequently observed RNA virus families, including *Flaviviridae*, *Rhabdoviridae*, *Iflaviridae*, *Nodaviridae*, *Mesoniviridae*, *Orthomyxoviridae* and *Totiviridae* (Supplementary Fig. S1C, discussed below). In addition, two DNA virus families were found in the top 10 of multiple mosquito genera, *Parvoviridae* and *Genomoviridae*.

Metagenomic surveys have the power to identify viruses of every organism present in the sample. Indeed, bacteriophages were frequently detected, predominantly from the tailed dsDNA bacteriophage families *Siphoviridae* [40,43,45,[61], [62], [63], [64]], *Myoviridae* [34,40,45,62,[64], [65], [66], [67], [68]], and *Autographiviridae* [40,65,67,69] (Fig. S1B)*.* These data underline that metagenomic sequencing can detect viral sequences of bacteria that colonize mosquitoes. Although it remains possible that mosquito physiology is affected by phage infection of bacterial symbionts [70], we have excluded phage families from further analyses.

**Table 1:** Mosquito genera in which viruses were detected with the corresponding number of species, continents, countries and studies. Sorted on the number of studies.

Genus	Species	Continents	Countries	Studies
Culex	33	6	42	114
Aedes	42	6	35	85
Anopheles	23	6	21	34
Mansonia	4	5	11	14
Armigeres	2	1	5	8
Coquillettidia	5	4	5	7
Culiseta	5	3	5	7
Psorophora	5	2	4	6
Ochlerotatus	3	4	5	5
Uranotaenia	1	2	2	3
Sabethes	2	1	1	2
Aedeomyia	1	1	1	1
Heamagogus	1	1	1	1
Wyeomyia	1	1	1	1

### 3.4 Positive-stranded RNA viruses

**Fig. 3:**
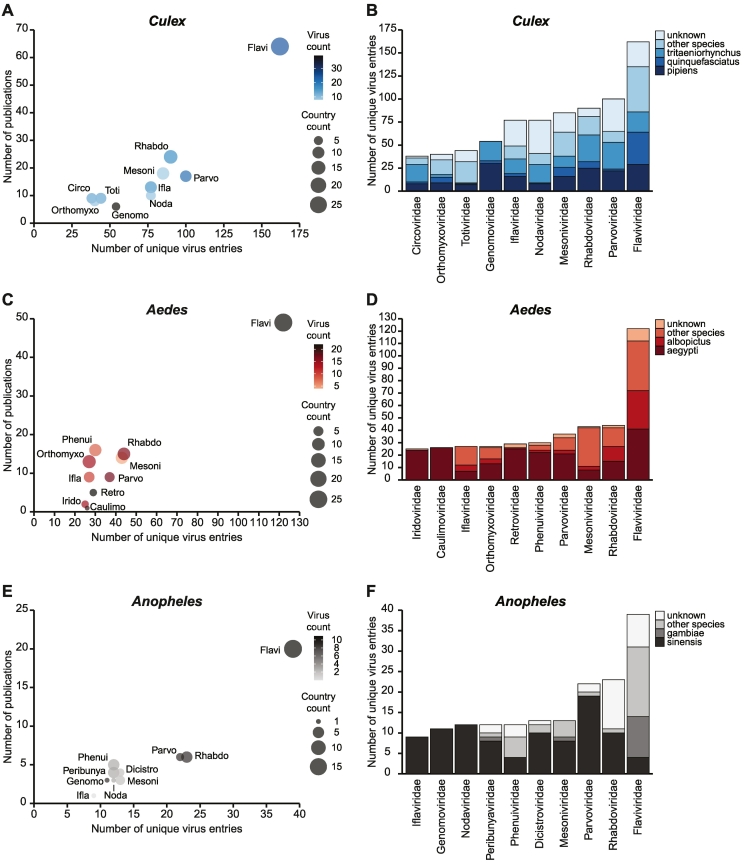
Most frequently reported virus families in mosquitoes. (A, C, E) Top 10 most frequently reported virus families for (A) *Culex*, (C) *Aedes*, and (E) *Anopheles* mosquitoes. The X-axis represents the number of unique virus entries for each family as a measure of virus abundance. The Y-axis indicates the number of studies that reported at least one virus from that family. Fill color indicates the total number of unique viruses detected for each family. Symbol size indicates the total number of countries for each virus family. (B, D, F) Top 10 most frequently reported virus families for (B) *Culex*, (D) *Aedes*, and (F) *Anopheles* mosquitoes with fill color indicating the mosquito species.

**Fig. 4:**
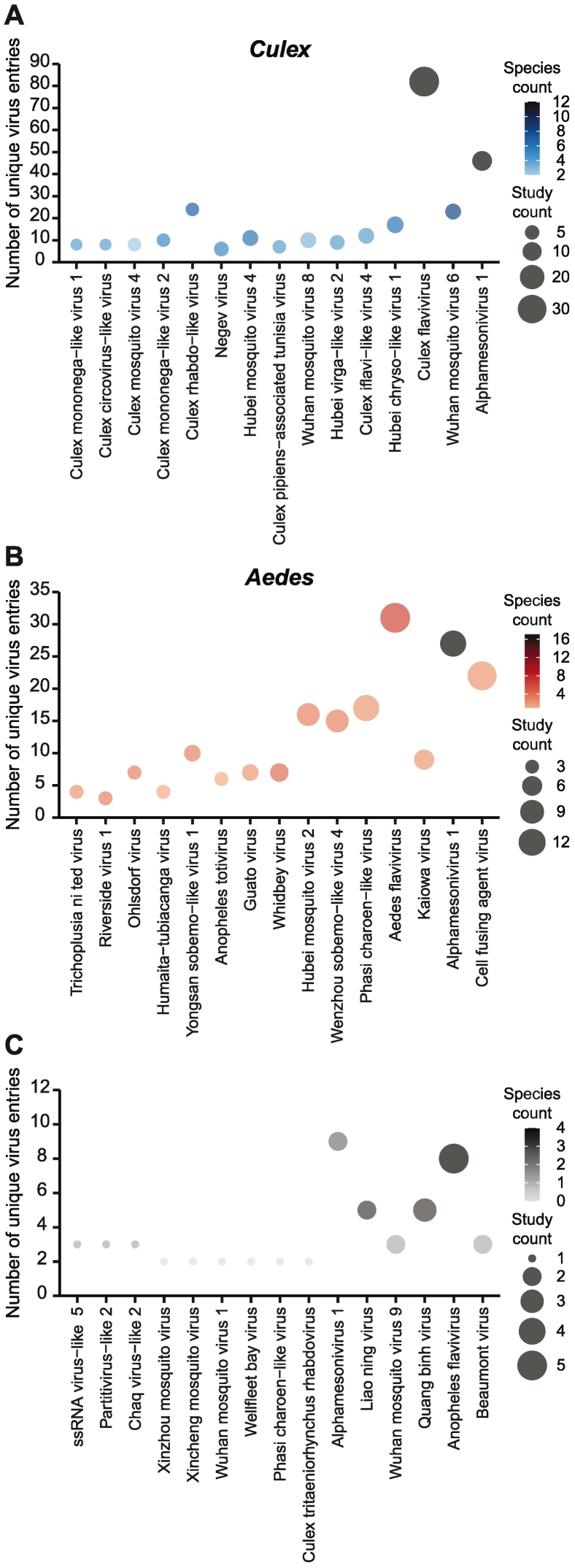
Most widespread mosquito viruses. Top 15 most widespread viruses for (A) *Culex*, (B) *Aedes*, and (C) *Anopheles* mosquitoes. Virus names were ordered according to the number of continents in which the virus was detected. Fill color indicates the number of mosquito species in which the virus has been found within the genus. Symbol size indicates the number of studies in which the virus was found.

We analyzed the contribution of virus families to the virome of *Culex, Aedes* and *Anopheles* mosquitoes, specifically. The *Flaviviridae* family*,* and specifically the *Flavivirus* genus, contains many mosquito-associated viruses including both arboviruses and ISVs [71,72]. In our dataset, *Flaviviridae* was the most abundant virus family in all mosquito genera (Fig. 3). A significant percentage of these entries derived from a few highly abundant viruses, including Culex flavivirus (51%) for the *Culex* genus, Aedes flavivirus (25%) and Cell fusing agent virus (20%) for the *Aedes* genus, and Anopheles flavivirus (23%) for the *Anopheles* genus. These viruses were among the most abundant and widespread in our dataset and have been detected in multiple species within and across mosquito genera (Fig. 4). Strikingly, Culex flavivirus seems to have a particularly broad host tropism, as it was detected in 12 species of *Culex* mosquitoes as well as three *Aedes* mosquito species and one species of *Anopheles* mosquitoes (Table 2). While flaviviruses are clearly highly prevalent, the family is overrepresented due to the frequent use of PCR studies to detect mosquito-associated flaviviruses, accounting for approximately 60% of *Flaviviridae* unique virus entries (Fig. S1C).

**Table 2:** Most widespread viruses in *Culex* mosquitoes.

Virus name	Virus family	Virus genus	Countries within each continent						Studies	Mosquito species	Unique entries
			Africa	Asia	Australia	Europe	North-America	South-America			
Alphamesonivirus 1	Mesoniviridae	Alphamesonivirus	–	2	1	3	2	1	11	12	46
Wuhan mosquito virus 6	Orthomyxoviridae	Quaranjavirus	–	1	1	2	1	1	6	8	23
Culex flavivirus	Flaviviridae	Flavivirus	2	6	–	–	3	4	33	12	82
Hubei chryso-like virus 1	Unclassified	Unclassified	–	2	1	2	1	–	7	6	17
Culex iflavi-like virus 4	Iflaviridae	Unclassified	–	1	–	2	1	1	6	4	12
Hubei virga-like virus 2	Unclassified	Unclassified	–	1	–	1	2	1	5	4	9
Wuhan mosquito virus 8	Chuviridae	Culicidavirus	–	1	–	1	1	1	6	3	10
Culex pipiens-associated tunisia virus	Unclassified	Unclassified	1	1	–	–	1	1	4	4	7
Hubei mosquito virus 4	Unclassified	Unclassified	–	2	–	–	1	1	6	6	11
Negev virus	Unclassified	Unclassified	–	1	–	1	–	1	5	5	6
Culex rhabdo-like virus	Rhabdoviridae	Ohlsrhavirus	–	1	1	–	1	–	4	7	24
Culex mononega-like virus 2	Unclassified	Unclassified	–	1	1	1	–	–	4	5	10
Culex mosquito virus 4	Chuviridae	Culicidavirus	–	1	–	1	1	–	4	2	8
Culex circovirus-like virus	Circoviridae	Circovirus	–	1	–	–	1	1	3	4	8
Culex mononega-like virus 1	Unclassified	Unclassified	–	1	1	1	–	–	3	4	8
Merida virus	Rhabdoviridae	Merhavirus	–	1	–	1	1	–	3	4	4
Culex bunyavirus 1	Unclassified	Unclassified	–	1	–	–	1	1	3	3	4
Wenzhou sobemo-like virus 3	Unclassified	Unclassified	–	3	–	2	–	–	7	4	19
Quang binh virus	Flaviviridae	Flavivirus	1	4	–	–	–	–	7	4	15
Hubei mosquito virus 2	Unclassified	Unclassified	–	3	–	1	–	–	6	4	17

The family of *Mesoniviridae* is a recently established taxon of mosquito-infecting positive-sense RNA viruses [17]. The large majority of *Mesoniviridae* virus entries in both *Culex* (74%) and *Aedes* (70%) mosquitoes were derived from alphamesonivirus 1, the founding species of the family that includes several closely related variants, such as Nam Dinh virus, Houston virus and Cavally virus [73,74]. Alphamesonivirus 1, with most entries from Nam Dinh virus and Houston virus, had a broad global distribution as it was detected in 11 countries across five continents (Table 2, Table 3). Strikingly, alphamesonivirus 1 also had the broadest host range of all viruses in our dataset, as it was detected in 34 mosquito species across five genera.

**Table 3:** Most widespread viruses in *Aedes* mosquitoes.

Virus name	Virus family	Virus genus	Countries within each continent						Studies	Mosquito species	Unique entries
			Africa	Asia	Australia	Europe	North-America	South-America			
Cell fusing agent virus	Flaviviridae	Flavivirus	3	4	1	–	3	1	14	2	22
Alphamesonivirus 1	Mesoniviridae	Alphamesonivirus	–	1	1	1	3	1	11	17	27
Kaiowa virus	Unclassified	Unclassified	–	2	1	1	1	1	6	2	9
Aedes flavivirus	Flaviviridae	Flavivirus	2	3	–	2	–	1	15	6	31
Phasi Charoen-like virus	Phenuiviridae	Phasivirus	–	3	1	–	3	1	11	2	17
Wenzhou sobemo-like virus 4	Unclassified	Unclassified	–	2	–	2	2	1	8	3	15
Hubei mosquito virus 2	Unclassified	Unclassified	–	2	–	2	1	1	8	3	16
Whidbey virus	Orthomyxoviridae	Unclassified	–	1	1	3	–	1	5	4	7
Guato virus	Unclassified	Unclassified	–	1	–	1	1	1	4	2	7
Anopheles totivirus	Totiviridae	Unclassified	–	1	1	–	1	1	3	1	6
Yongsan sobemo-like virus 1	Solemoviridae	Sobemovirus	–	2	–	1	–	1	4	3	10
Humaita-Tubiacanga virus	Unclassified	Unclassified	–	1	1	–	2	–	3	1	4
Ohlsdorf virus	Rhabdoviridae	Ohlsrhavirus	–	1	–	1	–	1	3	3	7
Riverside virus 1	Rhabdoviridae	Unclassified	–	1	–	1	–	1	3	3	3
Trichoplusia ni ted virus	Metaviridae	Errantivirus	–	1	–	–	1	1	3	2	4
Hubei toti-like virus 10	Unclassified	Unclassified	–	1	1	1	–	–	3	2	3
Menghai flavivirus	Flaviviridae	Flavivirus	–	1	–	–	1	1	3	2	3
dsRNA virus environmental sample	Unclassified	Unclassified	–	–	1	1	1	–	3	2	3
Croada virus	Unclassified	Unclassified	–	1	–	–	1	1	3	1	3
Blackford virus	Unclassified	Unclassified	–	1	1	–	1	–	2	1	3

*Nodaviridae* and *Iflaviridae* are both well-established families containing insect-associated viruses [75,76], which were frequently detected in *Culex* and *Aedes* mosquitoes. The majority of these virus entries was derived from only two studies, that sampled in California and China [77,78]. In China, *Nodaviridae* and *Iflaviridae* sequences, although not classified at the virus species level, were detected in 12 mosquito species [77]. In California, four iflaviruses (Culex iflavi-like virus 1–4) and five viruses currently classified as *Nodaviridae* (Culex Noda-like virus 1 and Culex mosquito virus 1, 2, 3 and 6) were detected in several species of *Culex* mosquitoes, representing 29% of the unique virus entries for *Iflaviridae* and 51% for *Nodaviridae* [78]. Culex iflavi-like virus 4 has also been detected in *Culex* mosquitoes in Brazil, China, Belgium and Serbia [65,[79], [80], [81]] (Table 2*)*.

### 3.5 Negative-stranded RNA viruses

Members of the *Rhabdoviridae* family were among the most frequently observed across mosquito genera in our dataset (Fig. 3) and included several of the most widespread viruses, including Culex rhabdo-like virus and Merida virus for *Culex* mosquitoes, Ohlsdorf virus and Riverside virus 1 for *Aedes* mosquitoes, and Beaumont virus and Wuhan mosquito virus 9 for *Anopheles* mosquitoes, which were each detected in at least three countries (Table 2, Table 3, Table 4). Notably, Merida virus has been found in four continents and in mosquito species of the genera *Culex*, *Aedes* and *Heamagogus* [32,33,40,66,79,82].

**Table 4:** Most widespread viruses in *Anopheles* mosquitoes.

Virus name	Virus family	Virus genus	Countries within each continent						Studies	Mosquito species	Unique entries
			Africa	Asia	Australia	Europe	North-America	South-America			
Beaumont virus	Rhabdoviridae	Unclassified	1	1	1	–	–	–	2	1	3
Anopheles flavivirus	Flaviviridae	Unclassified	5	–	–	1	–	–	5	4	8
Quang binh virus	Flaviviridae	Unclassified	2	1	–	–	–	–	3	3	5
Wuhan mosquito virus 9	Rhabdoviridae	Unclassified	1	2	–	–	–	–	2	1	3
Liao ning virus	Reoviridae	Seadornavirus	–	1	1	–	–	–	2	3	5
Alphamesonivirus 1	Mesoniviridae	Alphamesonivirus	–	1	1	–	–	–	2	2	9
Culex tritaeniorhynchus rhabdovirus	Rhabdoviridae	Merhavirus	1	1	–	–	–	–	1	n.d.⁎	2
Phasi Charoen-like virus	Phenuiviridae	Phasivirus	1	1	–	–	–	–	1	n.d.⁎	2
Wellfleet Bay virus	Orthomyxoviridae	Quaranjavirus	1	1	–	–	–	–	1	n.d.⁎	2
Wuhan mosquito virus 1	Phasmaviridae	Orthophasmavirus	1	1	–	–	–	–	1	n.d.⁎	2
Xincheng mosquito virus	Xinmoviridae	Anphevirus	1	1	–	–	–	–	1	n.d.⁎	2
Xinzhou mosquito virus	Peribunyaviridae	Unclassified	1	1	–	–	–	–	1	n.d.⁎	2
Chaq virus-like 2	Unclassified	Unclassified	3	–	–	–	–	–	1	1	3
Partitivirus-like 2	Partitiviridae	Unclassified	3	–	–	–	–	–	1	1	3
ssRNA virus-like 5	Unclassified	Unclassified	2	–	–	–	–	–	1	1	3
Bolahun virus	Xinmoviridae	Anphevirus	2	–	–	–	–	–	1	1	2
ssRNA virus-like 6	Unclassified	Unclassified	2	–	–	–	–	–	1	1	2
Chaq virus-like 3	Unclassified	Unclassified	2	–	–	–	–	–	1	1	2
Partitivirus-like 3	Partitiviridae	Unclassified	2	–	–	–	–	–	1	1	2
Hubei mosquito virus 2	Unclassified	Unclassified	–	1	–	–	–	–	3	1	4

⁎ n.d., *Anopheles* mosquitoes not defined at the species level.

The *Xinmoviridae* family was established in 2017 to encompass the free-floating genus *Anphevirus* in the *Mononegavirales* order [83]. Anpheviruses were detected in multiple mosquito species, including Xincheng mosquito virus and Bolahun virus in *Anopheles* mosquitoes and Aedes aegypti anphevirus, Aedes albopictus anphevirus and Aedes anphevirus in *Aedes* mosquitoes [66,79,81,84,85,86]. Notably, the contribution of *Xinmoviridae* to the mosquito virome may be underestimated due to the recent establishment of this family.

Several segmented viruses of the *Orthomyxoviridae* and *Phenuiviridae* were among the most widespread in *Culex* and *Aedes* mosquitoes (Fig. 4A,B). For the *Orthomyxoviridae*, these included Wuhan mosquito virus 6 for *Culex* mosquitoes and Whidbey virus for *Aedes* mosquitoes (Table 2, Table 3). In particular, Wuhan mosquito virus 6 showed a near-global distribution and broad mosquito host range, as it was detected in eight countries across all continents except Antarctica, and in 12 mosquito species across four genera (Supplementary file 1). *Phenuiviridae* was among the most frequently detected virus families in *Aedes* mosquitoes due to the high prevalence of Phasi Charoen-like virus, which contributed 60% of the entries of this family. Phasi Charoen-like virus is one of the most widespread viruses in *Aedes aegypti* specifically (Table 3), although it was also detected in *Aedes albopictus* [32]*, Culex quinquefasciatus* [86], *Haemagogus janthinomys* [82] and *Anopheles* mosquitoes [87].

### 3.6 Double-stranded RNA viruses

The *Artivirus* genus in the family *Totiviridae* is comprised of double-stranded RNA viruses of arthropods, including mosquitoes [9,88]. *Totiviridae* entries in our database corresponded to multiple totiviruses, with limited cross-detection between studies. Notably, the most frequently detected totivirus was Anopheles totivirus which, after its initial detection in *Anopheles gambiae* in Liberia [84], was found in *Aedes aegypti* in several countries across multiple continents [66,67,69] (Table 3).

### 3.7 DNA virus families

The *Parvoviridae* family of single-stranded DNA viruses is the most abundant DNA virus family in all three mosquito genera (Fig. 3). The family contains densoviruses, which have been studied as a potential biological control agent of insects and mosquitoes specifically [89]. A large proportion of the *Parvoviridae* entries for all three mosquito genera was derived from a single study [77], which detected parvovirus sequences in several *Culex*, *Aedes* and *Anopheles* species across China. An additional 38% of the *Parvoviridae* entries for *Culex* mosquitoes corresponded to Culex densovirus, which was detected in *Culex pipiens* and mixed pools of *Culex* mosquitoes across California [78].

Two families of circular Rep-encoding single-stranded DNA viruses (also referred to as CRESS-DNA viruses; [90]), *Genomoviridae* and *Circoviridae*, were frequently detected (Fig. 3; Fig. 4A). The high abundance of *Genomoviridae* in our dataset was mostly due to three metagenomic sequencing studies, each detecting sequences mapping to multiple genomoviruses [40,61,67]. For the *Circoviridae,* Culex circovirus-like virus was detected in three studies over three continents in species of all three genera [40,78,81].

For both *Genomoviridae* and *Circoviridae*, novel species have mostly been detected through metagenomic sequencing [91,92]. As active replication of these viruses has not been described in the animals sampled for sequencing, it is possible that these viruses are associated with food sources or pathogens of the host, precluding conclusions on the host range of these virus families [91,92]. However, for viruses from the *Circoviridae*, and specifically the *Cyclovirus* genus, arthropods (and mosquitoes specifically) have been suggested to be the primary host [67,93]. Moreover, Sclerotinia sclerotiorumhypovirulence-associated DNA virus 1, the founding species of the *Genomoviridae* viral family, was found to infect the mycophagous mosquito species *Lycoriella ingenua* under experimental conditions [94]*.* These studies suggest that vector mosquitoes could be a part of the host range of genomoviruses and circoviruses.

### 3.8 Unclassified viruses

The majority of virus entries in our database are unclassified at the family level (Fig. S1B). This large group included 114 unique virus entries from the genus *Negevirus* [18], a taxon of insect-specific, non-segmented, enveloped, positive-sense RNA viruses that has not been classified yet at the family level. Negeviruses are among the most abundant virus taxa in *Culex* (53 unique virus entries) and *Aedes* (32 unique virus entries) mosquitoes. Negeviruses have been reported in multiple *Culex* and *Aedes* species and across at least four continents. Among these, the most abundant virus was the eponymous Negev virus, which was detected in *Aedes aegypti* [81] and several *Culex* species across three continents [33,65,95,96] (Fig. 4A).

### 3.9 Most frequently detected viruses per mosquito genus

We collated lists of the top 20 most frequently detected viruses for each mosquito genus according to the number of continents and countries in which they were detected (Table 2, Table 3, Table 4, Fig. 4). For *Aedes* and *Culex*, the broad global distribution of these viruses was well supported, being detected in multiple countries in two to five continents in at least three independent studies, lending support to the validity of these observations. Surprisingly, all or nearly all top 20 viruses for *Culex* and *Aedes* respectively, were detected in multiple mosquito species, suggesting that vertical transmission is not the sole transmission route for these viruses. In line with the more limited sampling of *Anopheles* mosquitoes (Fig. 2), the top 20 *Anopheles* viruses were detected less frequently (between 2 and 9 unique virus entries) and at fewer places across the globe (Fig. 4C; Table 4).

A large number of unclassified viruses were among the top 20 (ten for *Culex,* nine for *Aedes*, and five for *Anopheles;* Table 2, Table 3, Table 4). Among these, some have a particularly broad global distribution, having been detected in at least four continents. These include Hubei chryso-like virus 1, Hubei virga-like virus 2 and Culex pipiens-associated Tunisia virus for *Culex* mosquitoes, and Kaiowa virus, Whenzhou Sobemo-like virus and Hubei mosquito virus 2 for *Aedes* mosquitoes (Table 2, Table 3). Notably, the detection of Kaiowa virus in *Aedes aegypti* metagenomic studies was proposed to be due to the presence of endogenous viral elements in mosquito genomes instead of replicating virus [71]. Metagenomic studies have also reported sequences with homology to Kaiowa virus in samples from *Aedes albopictus, Culex quinquefasciatus* and *Heamagogus janthinomys* [32,82,86,97] and the origin of these sequences merits further investigation.

## 4 Discussion

Growing scientific interest and increasing accessibility to deep-sequencing technology has led to a large body of literature on the mosquito virome. We have collated information from 175 research articles from the last 22 years to construct a comprehensive and searchable database of mosquito-associated viruses, along with the locations and hosts in which they have been detected. We found that RNA viruses from the families *Flaviviridae* and *Rhabdoviridae* are widespread in *Culex, Aedes* and *Anopheles* mosquitoes globally. We collated lists of the top 20 viruses with the widest global distribution for each of these mosquito genera and found that most of these viruses were detected in multiple mosquito species within, and sometimes across mosquito genera. The prevalence and overall stability of these viruses within mosquito populations and the transmission routes enabling them to persist and spread merits further investigation.

We collated virome data as reported, accepting the authors' assessment for assigning viral sequences to established virus species or taxons or for considering a virus novel. A limitation of this approach is that different thresholds for genome coverage, number of virus mapping reads or contigs, and nucleotide or amino acid sequence identity scores were used for virus identification between studies. More concerningly, some studies did not unambiguously report the criteria used for virus identification, which makes side-by-side comparisons of studies difficult. Consequently, the database contains some low-confidence virus entries due to insufficiently stringent thresholds for virus identification or misclassification of virus sequences. Due to these caveats and differences in sampling intensities, our database cannot be used to accurately infer the absence of a virus in specific mosquito species. Another limitation of our study is that we may have inevitably missed relevant articles that did not match our search terms, despite our best efforts to use a comprehensive literature search strategy. Despite these limitations, the most widespread and abundant viruses in our dataset have been found in multiple independent studies. As such, the collated top 20 most widespread viruses can be considered high-confidence constituents of the mosquito virome, especially for *Aedes* and *Culex* that have been sampled most extensively.

During our study, we noticed that virome data are often impractically reported for interpretation and re-use by other scientists, due to unreported critical variables or an impractical format to present results (e.g., in heat-maps) without accompanying presentation in a reusable data format. We propose that reporting can be improved by the standard inclusion of a supplementary table containing per virus positive sample, *i)* the viruses identified along with accession numbers, nucleotide and amino acid identity scores, genome coverage, numbers of reads/contigs mapping to the viral genome, *ii)* information on the sample, such as pool identifier, number of mosquitoes per sample, mosquito species, sex, and life stage, *iii)* the date and location of sampling, along with geographic coordinates and type of habitat, and *iv)* sequencing information, including the sequencing platform, method for library preparation, sequencing depth per library, and accession number of the repository in which the raw sequence data have been deposited.

Next-generation sequencing is a relatively unbiased approach that successfully detects both RNA and DNA viruses in mosquitoes. However, the detection of sequences of well-known mammalian viruses of the *Retroviridae* [40,65,66,82] (e.g., murine leukemia virus), *Herpesviridae* [40,45,65] (e.g., herpes simplex virus) and *Hepadnaviridae* (e.g., hepatitis B virus) [61] indicates that some datasets contain considerable amount of noise. The origin of these sequences is unclear but may be due to insufficiently stringent thresholds for virus identification, to biological contaminants such as sequences derived from blood meals, or to experimental contamination during library preparation and sequencing. Alternatively, although no insect viruses have currently been described in the family *Retroviridae*, it remains possible that these sequences derive from unidentified mosquito retroviruses or from retroelements in the mosquito genome.

Detection of viral sequences does not provide direct support of active replication in the mosquito host and, indeed, bacteriophage sequences were frequently detected in mosquito virome studies [34,40,43,45,65,67,69]. Isolation of a virus in mosquito cell culture would provide strong support for active virus replication in the mosquito host [98,99]. Alternatively, small RNA-sequencing approaches may be used to distinguish sequences of actively replicating viruses from contaminating sequences. Viral double-stranded RNA produced during replication of both DNA and RNA viruses are processed into 21-nt small interfering RNAs [100] that can be readily distinguished in small RNA size profiles. Indeed, some researchers have used small RNA sequencing as an alternative or complement to conventional mRNA sequencing for virome studies in insects [[101], [102], [103], [104]].

Small RNAs may also help to distinguish replicating viruses from another source of viral sequences, EVEs. The *Aedes aegypti* and *Aedes albopictus* genomes contain a large number of non-retroviral EVEs [[105], [106], [107]], transcripts of which may be detected in RNA-seq experiments. Next-generation sequencing studies therefore require careful analysis to differentiate between EVE-derived sequences and virus-derived sequences. The removal of contigs mapping to mosquito genomes may be impossible for species lacking reference genomes and, even for species with high-quality reference genomes, this may be insufficient as the EVE repertoire differs between mosquito populations [108]. Small RNA sequencing may help to distinguish EVE-derived sequences from sequences of replicating viruses as EVEs may primarily give rise to PIWI-interacting RNAs (piRNAs) with a typical size of 25–30 nt and strong strand biases. These can be readily distinguished from replication-dependent siRNAs of 21 nt, which are usually derived from both positive and negative-sense viral RNAs [102,108].

The composition of the mosquito virome is likely shaped by the environment, virus transmission modes, and restrictive factors in specific mosquito species or genera. Moreover, changing biotic and abiotic factors associated with global warming and increasing globalization may further affect the mosquito virome. Our database is an up-to-date, comprehensive overview of primary literature on mosquito-associated viruses from the last 22 years. As such, our study forms a solid foundation to study inter- and intra-species pathogen transmission from a One Health perspective. A future challenge will be to understand how virome dynamics affect mosquito-borne disease outbreaks.

## Note added in proof

In agreement with our analyses, Olmo *et al.* recently reported that Phasi Charoen-like virus and Humaita Tubiacanga virus were highly abundant and widespread in *Ae. aegypti* worldwide (Nat Microbiol. 2023, 8:135-149).

The following are the supplementary data related to this article.

Supplementary file 1 Final selection of 175 articles used for the analyses and their references. Supplementary file 1

Supplementary Table S1 Sample-structured database of viruses identified in mosquito virome studies. Supplementary Table S1

Supplementary Table S2 Virus positive mosquito species, with the corresponding number of continents, countries and studies in which they are sampled. Supplementary Table S2

Supplementary Fig. S1 Unique virus entries in mosquito virome studies. (A) Number of unique virus entries reported for the top 50 studies that contributed most entries in our database. Unique virus entries for Hameed et al. (2020) before (dark red) and after (salmon) data curation are indicated. (B) Number of unique virus entries corresponding to the individual virus families for all mosquito species in our database. Bacteriophage families are indicated with salmon fill color; viruses not classified at the family level in light blue. (C) Number of unique virus entries for the top 10 most frequently detected families, according to detection method. Supplementary Fig. S1

## Declaration of Competing Interest

The authors declare that they have no known competing financial interests or personal relationships that could have appeared to influence the work reported in this paper.

## Data Availability

This is a review of published literature. The data are available in the supplementary files.
