# Supplementary material for: Composition and global distribution of the mosquito virome - A comprehensive database of insect-specific viruses
Source: One Health. 2023 Jan 20;16:100490. doi: 10.1016/j.onehlt.2023.100490 (PMC9929601; doi:10.1016/j.onehlt.2023.100490)
Supplement: Supplementary file 1 — Final selection of 175 articles used for the analyses and their references. [file mmc1.pdf]

Supplementary file 1. Final selection of 175 articles used for the analyses and their references

Study	Reference
Abilio_BMCMicrobiol_2020	[1]
Ajamma_PLoSNeglTropDis_2018	[2]
Ali_Nature_2021	[3]
Atoni_Viruses_2018	[4]
Auguste_JGenVirol_2014	[5]
Auguste_Virology_2021	[6]
Bahk_KoreanJParasitol_2020	[7]
Baidaliuk_VirusEvolution_2020	[8]
Batson_eLife_2021	[9]
Belda_BMCGenomics_2019	[10]
Bennouna_Virology_2019	[11]
Bigot_VirusEvol_2018	[12]
Birnberg_Viruses_2020	[13]
Blitvich_JMedEntomol_2010	[14]
Bolling_AmJTropMedHyg_2011	[15]
Carissimo_PLoSOne_2016	[16]
Carrera_AmJTropMedHyg_2015	[17]
Chandler_frontiersinMicrobiology_2015	[18]
Chandler_Virology_2014	[19]
Charles_JournalofGeneralVirology_2018	[20]
Chatterjee_VirolJ_2021	[21]
Chen_CompImmunolMicrobiolInfectDis_2013	[22]
Chiuya_PLosOne_2021	[23]
Cholleti_PLOSONE_2016	[24]
Cholleti_VirolJ_2018	[25]
Coffey_Virology_2014	[26]
Contreras-Gutierrez_Virology_2017	[27]
Cook_PLOSONE_2013	[28]
Crabtree_ArchVirol_2009	[29]
Cunha_ActaTropica_2020	[30]
Cunha_MicrobiolResourAnnounc_2020	[31]
daSilva_VirusResearch_2021	[32]
daSilvaFerreira_InfectionGeneticsandEvolution_2020	[33]
daSilvaNeves_VirusResearch_2021	[34]
daSilvaRibeiro_VirologyJournal_2022	[35]
Datta_Intervirology_2015	[36]
deAraujo_ArchVirol_2012	[37]
deOliveira_SciRep_2021	[38]
Diagne_VirusRes_2020	[39]
Du_Life.scichina_2020	[40]
Duarte_BrazilianJournalofMicrobiology_2021	[41]
Ergunay_ArchVirol_2017	[42]

Ergunay_InfectGenetEvol_2016	[43]
Ergünay_Parasites&Vectors_2017	[44]
Evangelina_JInvertebrPathol_2013	[45]
Faizah_Viruses_2020	[46]
Fan_ArchVirol_2016	[47]
Fang_ApplEnvironMicrobiol_2021	[48]
Fang_FrontCellInfectMicrobiol_2021	[49]
Fang_InfectDisPoverty_2018	[50]
Fang_InfectiousDiseasesofPoverty_2021	[51]
Farfan-Ale_ AmJTropMedHyg_2009	[52]
Farfan-Ale_VectorBorneZoonoticDis_2010	[53]
Fauver_Virology_2016	[54]
Fernandes_ActaTrop_2016	[55]
Fernandes_Viruses_2018	[56]
Ferreira_VirusGenes_2013	[57]
Frey_EvolutionaryBioinformatics_2016	[58]
Fujita_ArchVirol_2017	[59]
Garigliany_JGenVirol_2015	[60]
Gil_MolEcolResour_2021	[61]
Gravina_Virology_2019	[62]
Grisenti_JGenVirol_2015	[63]
Guarido_Viruses_2021	[64]
Guggemos_PLoSNeglTropDis_2021	[65]
Guzman_AmJTropMedHyg_2018	[66]
Hameed_FrontMicrobiol_2020	[67]
Hameed_TransboundEmergDisease_2020	[68]
Harrison_mSphere_2020	[69]
Harrison_Viruses_2016	[70]
He_ParasitVectors_2021	[71]
He_PLosNeglTropDis_2021	[72]
Hermanns_JGenVirol_2017	[73]
Hermanns_JGenVirol_2020	[74]
Hobson-Peters_PLoSOne_2013	[75]
Hobson-Peters_Virology_2016	[76]
Hoshino_Virology_2009	[77]
Huang_JInvertebrPathol_2015	[78]
Huanyu_VirolJ_2012	[79]
Huhtamo_Virology_2012	[80]
Huhtamo_Virology_2014	[81]
Iwashita_TropMedHealth_2018	[82]
Jeffries_WellcomeOpenRes_2020	[83]
Kallies_Viruses_2014	[84]
Kawakami_ArchVirol_2016	[85]
Kenney_JGenVirol_2014	[86]
Kim_Virology_2009	[87]

Klein_JMedEntomol_2021	[88]
Kobayashi_JGenVirol_2017	[89]
Kopp_Viruses_2019	[90]
Kubacki_Viruses_2020	[91]
Kuwata_ArchVirol_2013	[92]
Kuwata_ArchVirol_2015	[93]
KyawKyaw_VirusRes_2018	[94]
LaraPinto_PloSOne_2017	[95]
Liang_ArchVirol_2015	[96]
Liu_BriefBioinform_2021	[97]
Ma_PloSOne_2011	[98]
Machado_Intervirology_2012	[99]
Maia_Viruses_2019	[100]
Marklewitz_ProcNatlAcadSciUSA_2015	[101]
Martin_ApplEnvironMicrobiol_2019	[102]
Martin_Arch Virol_2020	[103]
McLean_Virology_2015	[104]
Miranda_Virol J_2019	[105]
Misencik_Vector Borne Zoonotic Dis_2016	[106]
Moraes_Med Vet Entomol_2019	[107]
Morais_Infect Genet Evol_2020	[108]
Munivenkatappa_VectorBorneZoonoticDis_2021	[109]
Muttis_ArchVirol_2012	[110]
Nabeshima_JGenVirol_2014	[111]
Nebbak_Viruses_2021	[112]
Newton_Viruses_2020	[113]
Ng_PLoSOne_2011	[114]
Obara-Nagoya_JMedEntomol_2013	[115]
Obrien_EvolBioinformOnline_2017	[116]
Obrien_Viruses_2020	[117]
Öhlund_Viruses_2019	[118]
Öncü_Infect Genet Evol_2018	[119]
Orba_JGenVirol_2021	[120]
Papa_Arch Virol_2016	[121]
Parreira_VirusRes_2012	[122]
Parry_Viruses_2020	[123]
Pettersson_Viruses_2019	[124]
Pyke_VirusEvol_2021	[125]
Ramírez_mSphere_2020	[126]
Ramos-Nino_PLoSOne_2020	[127]
Ramos-Nino2_PLoSOne_2020	[128]
Reuter_InfectGenetEvol_2016	[129]
Ribeiro_Insects_2019	[130]
Ribeiro_Viruses_2019	[131]
Ribeiro_Viruses_2020	[132]

Rizzo_ParasitVectors_2014	[133]
Rwegoshora_JTropMed_2000	[134]
Sadeghi_Virology_2018	[135]
Sadeghi_VirusRes_2017	[136]
Sanborn_MolEcol_2021	[137]
Sanborn_Viruses_2019	[138]
Scarpassa_BMCGenomics_2019	[139]
Schuster_Jvirol_2014	[140]
Shahhosseini_InfectGenetEvol_2017	[141]
Shi_JVirol_2017	[142]
Shi_Microbiome_2019	[143]
Shi_mSystems_2020	[144]
Shi_PLoSOne_2015	[145]
Silva_VirusRes_2019	[146]
Stanojevic_Viruses_2020	[147]
Supriyono_JVetMedSci_2020	[148]
Suvanto_ArchVirol_2020	[149]
Takhampunya_JMedEntomol_2014	[150]
Tangudu_JGenVirol_2019	[151]
Tesh_Viruses_2020	[152]
Thannesberger_Pathogens_2020	[153]
Thongsripong_SciRep_2021	[154]
Tyler_AmJTropMedHyg_2011	[155]
Vasilakis_VirolJ_2014	[156]
Villinger_MolEcolResour_2017	[157]
Wang_ArchVirol_2019	[158]
Wang_mSphere_2021	[159]
Warrilow_PLoSOne_2018	[160]
Wastika_Viruses_2020	[161]
Williams_PLoSOne_2020	[162]
Williams_Viruses_2020	[163]
Xia_VirolSin_2018	[164]
Xiao_FrontCellInfectMicrobiol_2018	[165]
Yamao_ArchVirol_2009	[166]
Yezli_IntJEnvironResPublicHealth_2021	[167]
Zakrzewski_SciRep_2018	[168]
Zana_ActaVirol_2017	[169]
Zhai_JGenVirol_2008	[170]
Zhang_ArchVirol_2017	[171]
Zhang_Virology_2018	[172]
Zhao_FrontMicrobiol_2019	[173]
Zhao_SciTotalEnviron_2022	[174]
Zuo_VirusRes_2014	[175]

References

1. Abílio, A.P., et al., *A survey of RNA viruses in mosquitoes from Mozambique reveals novel genetic lineages of flaviviruses and phenuiviruses, as well as frequent flavivirus-like viral DNA forms in Mansonia.* BMC Microbiol, 2020. **20**(1): p. 225.
2. Ajamma, Y.U., et al., *Vertical transmission of naturally occurring Bunyamwera and insect-specific flavivirus infections in mosquitoes from islands and mainland shores of Lakes Victoria and Baringo in Kenya.* PLoS Negl Trop Dis, 2018. **12**(11): p. e0006949.
3. Ali, R., et al., *Characterization of the virome associated with Haemagogus mosquitoes in Trinidad, West Indies.* Sci Rep, 2021. **11**(1): p. 16584.
4. Atoni, E., et al., *Metagenomic Virome Analysis of Culex Mosquitoes from Kenya and China.* Viruses, 2018. **10**(1).
5. Auguste, A.J., et al., *Characterization of a novel Negevirus and a novel Bunyavirus isolated from Culex (Culex) declarator mosquitoes in Trinidad.* J Gen Virol, 2014. **95**(Pt 2): p. 481-485.
6. Auguste, A.J., et al., *Isolation of a novel insect-specific flavivirus with immunomodulatory effects in vertebrate systems.* Virology, 2021. **562**: p. 50-62.
7. Bahk, Y.Y., et al., *Monitoring Culicine Mosquitoes (Diptera: Culicidae) as a Vector of Flavivirus in Incheon Metropolitan City and Hwaseong-Si, Gyeonggi-Do, Korea, during 2019.* Korean J Parasitol, 2020. **58**(5): p. 551-558.
8. Baidaliuk, A., et al., *Novel genome sequences of cell-fusing agent virus allow comparison of virus phylogeny with the genetic structure of Aedes aegypti populations.* Virus Evol, 2020. **6**(1): p. veaa018.
9. Batson, J., et al., *Single mosquito metatranscriptomics identifies vectors, emerging pathogens and reservoirs in one assay.* Elife, 2021. **10**.
10. Belda, E., et al., *De novo profiling of RNA viruses in Anopheles malaria vector mosquitoes from forest ecological zones in Senegal and Cambodia.* BMC Genomics, 2019. **20**(1): p. 664.
11. Bennouna, A., et al., *Identification of Eilat virus and prevalence of infection among Culex pipiens L. populations, Morocco, 2016.* Virology, 2019. **530**: p. 85-88.
12. Bigot, D., et al., *Discovery of Culex pipiens associated tunisia virus: a new ssRNA(+) virus representing a new insect associated virus family.* Virus Evol, 2018. **4**(1): p. vex040.
13. Birnberg, L., et al., *Viromics on Honey-Baited FTA Cards as a New Tool for the Detection of Circulating Viruses in Mosquitoes.* Viruses, 2020. **12**(3).
14. Blitvich, B.J., et al., *Genomic sequence and phylogenetic analysis of Culex flavivirus, an insect-specific flavivirus, isolated from Culex pipiens (Diptera: Culicidae) in Iowa.* J Med Entomol, 2009. **46**(4): p. 934-41.
15. Bolling, B.G., et al., *Insect-specific flaviviruses from Culex mosquitoes in Colorado, with evidence of vertical transmission.* Am J Trop Med Hyg, 2011. **85**(1): p. 169-77.
16. Carissimo, G., et al., *Identification and Characterization of Two Novel RNA Viruses from Anopheles gambiae Species Complex Mosquitoes.* PLoS One, 2016. **11**(5): p. e0153881.
17. Carrera, J.P., et al., *Mercadeo Virus: A Novel Mosquito-Specific Flavivirus from Panama.* Am J Trop Med Hyg, 2015. **93**(5): p. 1014-9.
18. Chandler, J.A., R.M. Liu, and S.N. Bennett, *RNA shotgun metagenomic sequencing of northern California (USA) mosquitoes uncovers viruses, bacteria, and fungi.* Front Microbiol, 2015. **6**: p. 185.
19. Chandler, J.A., et al., *Metagenomic shotgun sequencing of a Bunyavirus in wild-caught Aedes aegypti from Thailand informs the evolutionary and genomic history of the Phleboviruses.* Virology, 2014. **464-465**: p. 312-319.
20. Charles, J., et al., *Detection of novel and recognized RNA viruses in mosquitoes from the Yucatan Peninsula of Mexico using metagenomics and characterization of their in vitro host ranges.* J Gen Virol, 2018. **99**(12): p. 1729-1738.

21. Chatterjee, S., et al., *Molecular detection and identification of Culex flavivirus in mosquito species from Jeju, Republic of Korea.* Virol J, 2021. **18**(1): p. 150.
22. Chen, Y.Y., et al., *First detection of the Africa/Caribbean/Latin American subtype of Culex flavivirus in Asian country, Taiwan.* Comp Immunol Microbiol Infect Dis, 2013. **36**(4): p. 387-96.
23. Chiuya, T., et al., *A survey of mosquito-borne and insect-specific viruses in hospitals and livestock markets in western Kenya.* PLoS One, 2021. **16**(5): p. e0252369.
24. Cholleti, H., et al., *Discovery of Novel Viruses in Mosquitoes from the Zambezi Valley of Mozambique.* PLoS One, 2016. **11**(9): p. e0162751.
25. Cholleti, H., et al., *Genetic characterization of a novel picorna-like virus in Culex spp. mosquitoes from Mozambique.* Virol J, 2018. **15**(1): p. 71.
26. Coffey, L.L., et al., *Enhanced arbovirus surveillance with deep sequencing: Identification of novel rhabdoviruses and bunyaviruses in Australian mosquitoes.* Virology, 2014. **448**: p. 146-58.
27. Contreras-Gutiérrez, M.A., et al., *Sinu virus, a novel and divergent orthomyxovirus related to members of the genus Thogotovirus isolated from mosquitoes in Colombia.* Virology, 2017. **501**: p. 166-175.
28. Cook, S., et al., *Novel virus discovery and genome reconstruction from field RNA samples reveals highly divergent viruses in dipteran hosts.* PLoS One, 2013. **8**(11): p. e80720.
29. Crabtree, M.B., P.T. Nga, and B.R. Miller, *Isolation and characterization of a new mosquito flavivirus, Quang Binh virus, from Vietnam.* Arch Virol, 2009. **154**(5): p. 857-60.
30. Cunha, M.S., et al., *Detection and characterization of Ilheus and Iguape virus genomes in historical mosquito samples from Southern Brazil.* Acta Trop, 2020. **205**: p. 105401.
31. Cunha, M.D.P., et al., *A Metagenomic Approach Identified a Novel Phasi Charoen-Like Virus Coinfecting a Chikungunya Virus-Infected Aedes aegypti Mosquito in Brazil.* Microbiol Resour Announc, 2020. **9**(31).
32. da Silva, A.F., et al., *Metatranscriptomic analysis identifies different viral-like sequences in two neotropical Mansoniini mosquito species.* Virus Res, 2021. **301**: p. 198455.
33. da Silva Ferreira, R., et al., *Insect-specific viruses and arboviruses in adult male culicids from Midwestern Brazil.* Infect Genet Evol, 2020. **85**: p. 104561.
34. da Silva Neves, N.A., et al., *Sialovirome of Brazilian tropical anophelines.* Virus Res, 2021. **302**: p. 198494.
35. da Silva Ribeiro, A.C., et al., *Negeviruses isolated from mosquitoes in the Brazilian Amazon.* Virol J, 2022. **19**(1): p. 17.
36. Datta, S., et al., *Phylogenetic Characterization of a Novel Insect-Specific Flavivirus Detected in a Culex Pool, Collected from Assam, India.* Intervirology, 2015. **58**(3): p. 149-54.
37. de Araujo Coutinho, C.J., et al., *Occurrence and phylogenetic characterization of a baculovirus isolated from Culex quinquefasciatus in São Paulo State, Brazil.* Arch Virol, 2012. **157**(9): p. 1741-5.
38. de Oliveira Ribeiro, G., et al., *Guapiaçu virus, a new insect-specific flavivirus isolated from two species of Aedes mosquitoes from Brazil.* Sci Rep, 2021. **11**(1): p. 4674.
39. Diagne, M.M., et al., *Dianke virus: A new mesonivirus species isolated from mosquitoes in Eastern Senegal.* Virus Res, 2020. **275**: p. 197802.
40. Du, J., et al., *Characterization of viromes within mosquito species in China.* Sci China Life Sci, 2020. **63**(7): p. 1089-1092.
41. Duarte, M.A., et al., *Identification of potential new mosquito-associated viruses of adult Aedes aegypti mosquitoes from Tocantins state, Brazil.* Braz J Microbiol, 2021.
42. Ergünay, K., et al., *A novel rhabdovirus, related to Merida virus, in field-collected mosquitoes from Anatolia and Thrace.* Arch Virol, 2017. **162**(7): p. 1903-1911.

43. Ergünay, K., et al., *Isolation and genomic characterization of Culex theileri flaviviruses in field-collected mosquitoes from Turkey.* Infect Genet Evol, 2016. **46**: p. 138-147.
44. Ergünay, K., et al., *Co-circulation of West Nile virus and distinct insect-specific flaviviruses in Turkey.* Parasit Vectors, 2017. **10**(1): p. 149.
45. Evangelina, M., M.M. Victoria, and G.J. José, *Culex pipiens affected by joint infection of a mosquito iridescent virus and Strelkovimermis spiculatus.* J Invertebr Pathol, 2013. **114**(3): p. 295-7.
46. Faizah, A.N., et al., *Deciphering the Virome of Culex vishnui Subgroup Mosquitoes, the Major Vectors of Japanese Encephalitis, in Japan.* Viruses, 2020. **12**(3).
47. Fan, H., et al., *Complete genome sequence of Xishuangbanna flavivirus, a novel mosquito-specific flavivirus from China.* Arch Virol, 2016. **161**(6): p. 1723-7.
48. Fang, Y., et al., *Detection of DENV-2 and Insect-Specific Flaviviruses in Mosquitoes Collected From Jeddah, Saudi Arabia.* Front Cell Infect Microbiol, 2021. **11**: p. 626368.
49. Fang, Y., et al., *Monitoring Mosquito-Borne Arbovirus in Various Insect Regions in China in 2018.* Front Cell Infect Microbiol, 2021. **11**: p. 640993.
50. Fang, Y., et al., *Co-circulation of Aedes flavivirus, Culex flavivirus, and Quang Binh virus in Shanghai, China.* Infect Dis Poverty, 2018. **7**(1): p. 75.
51. Fang, Y., et al., *Molecular epidemiology of mosquito-borne viruses at the China-Myanmar border: discovery of a potential epidemic focus of Japanese encephalitis.* Infect Dis Poverty, 2021. **10**(1): p. 57.
52. Farfan-Ale, J.A., et al., *Detection of RNA from a novel West Nile-like virus and high prevalence of an insect-specific flavivirus in mosquitoes in the Yucatan Peninsula of Mexico.* Am J Trop Med Hyg, 2009. **80**(1): p. 85-95.
53. Farfan-Ale, J.A., et al., *Detection of flaviviruses and orthobunyaviruses in mosquitoes in the Yucatan Peninsula of Mexico in 2008.* Vector Borne Zoonotic Dis, 2010. **10**(8): p. 777-83.
54. Fauver, J.R., et al., *West African Anopheles gambiae mosquitoes harbor a taxonomically diverse virome including new insect-specific flaviviruses, mononegaviruses, and totiviruses.* Virology, 2016. **498**: p. 288-299.
55. Fernandes, L.N., et al., *Detection of Culex flavivirus and Aedes flavivirus nucleotide sequences in mosquitoes from parks in the city of São Paulo, Brazil.* Acta Trop, 2016. **157**: p. 73-83.
56. Fernandes, L.N., et al., *A Novel Highly Divergent Strain of Cell Fusing Agent Virus (CFAV) in Mosquitoes from the Brazilian Amazon Region.* Viruses, 2018. **10**(12).
57. Ferreira, D.D., et al., *Characterization of an insect-specific flavivirus (OCFVPT) co-isolated from Ochlerotatus caspius collected in southern Portugal along with a putative new Negev-like virus.* Virus Genes, 2013. **47**(3): p. 532-45.
58. Frey, K.G., et al., *Bioinformatic Characterization of Mosquito Viromes within the Eastern United States and Puerto Rico: Discovery of Novel Viruses.* Evol Bioinform Online, 2016. **12**(Suppl 2): p. 1-12.
59. Fujita, R., et al., *Bustos virus, a new member of the negevirus group isolated from a Mansonia mosquito in the Philippines.* Arch Virol, 2017. **162**(1): p. 79-88.
60. Garigliany, M.M., et al., *Characterization of a novel circo-like virus in Aedes vexans mosquitoes from Germany: evidence for a new genus within the family Circoviridae.* J Gen Virol, 2015. **96**(Pt 4): p. 915-920.
61. Gil, P., et al., *A library preparation optimized for metagenomics of RNA viruses.* Mol Ecol Resour, 2021. **21**(6): p. 1788-1807.
62. Gravina, H.D., et al., *Identification of insect-specific flaviviruses in areas of Brazil and Paraguay experiencing endemic arbovirus transmission and the description of a novel flavivirus infecting Sabethes belisarioi.* Virology, 2019. **527**: p. 98-106.
63. Grisenti, M., et al., *Wide detection of Aedes flavivirus in north-eastern Italy--a European hotspot of emerging mosquito-borne diseases.* J Gen Virol, 2015. **96**(Pt 2): p. 420-430.

64. Guarido, M.M., et al., *Detection of Insect-Specific Flaviviruses in Mosquitoes (Diptera: Culicidae) in Northeastern Regions of South Africa.* Viruses, 2021. **13**(11).
65. Guggemos, H.D., et al., *Simultaneous circulation of two West Nile virus lineage 2 clades and Bagaza virus in the Zambezi region, Namibia.* PLoS Negl Trop Dis, 2021. **15**(4): p. e0009311.
66. Guzman, H., et al., *Characterization of Three New Insect-Specific Flaviviruses: Their Relationship to the Mosquito-Borne Flavivirus Pathogens.* Am J Trop Med Hyg, 2018. **98**(2): p. 410-419.
67. Hameed, M., et al., *A Metagenomic Analysis of Mosquito Virome Collected From Different Animal Farms at Yunnan-Myanmar Border of China.* Front Microbiol, 2020. **11**: p. 591478.
68. Hameed, M., et al., *A viral metagenomic analysis reveals rich viral abundance and diversity in mosquitoes from pig farms.* Transbound Emerg Dis, 2020. **67**(1): p. 328-343.
69. Harrison, J.J., et al., *Antigenic Characterization of New Lineage II Insect-Specific Flaviviruses in Australian Mosquitoes and Identification of Host Restriction Factors.* mSphere, 2020. **5**(3).
70. Harrison, J.J., et al., *A New Orbivirus Isolated from Mosquitoes in North-Western Australia Shows Antigenic and Genetic Similarity to Corriparta Virus but Does Not Replicate in Vertebrate Cells.* Viruses, 2016. **8**(5).
71. He, W., et al., *Virome in adult Aedes albopictus captured during different seasons in Guangzhou City, China.* Parasit Vectors, 2021. **14**(1): p. 415.
72. He, X., et al., *Metagenomic sequencing reveals viral abundance and diversity in mosquitoes from the Shaanxi-Gansu-Ningxia region, China.* PLoS Negl Trop Dis, 2021. **15**(4): p. e0009381.
73. Hermanns, K., et al., *Discovery of a novel alphavirus related to Eilat virus.* J Gen Virol, 2017. **98**(1): p. 43-49.
74. Hermanns, K., et al., *Agua Salud alphavirus defines a novel lineage of insect-specific alphaviruses discovered in the New World.* J Gen Virol, 2020. **101**(1): p. 96-104.
75. Hobson-Peters, J., et al., *A new insect-specific flavivirus from northern Australia suppresses replication of West Nile virus and Murray Valley encephalitis virus in co-infected mosquito cells.* PLoS One, 2013. **8**(2): p. e56534.
76. Hobson-Peters, J., et al., *Discovery and characterisation of a new insect-specific bunyavirus from Culex mosquitoes captured in northern Australia.* Virology, 2016. **489**: p. 269-81.
77. Hoshino, K., et al., *Isolation and characterization of a new insect flavivirus from Aedes albopictus and Aedes flavopictus mosquitoes in Japan.* Virology, 2009. **391**(1): p. 119-29.
78. Huang, Y., et al., *Isolation and characterization of a novel invertebrate iridovirus from adult Anopheles minimus (AMIV) in China.* J Invertebr Pathol, 2015. **127**: p. 1-5.
79. Huanyu, W., et al., *Isolation and identification of a distinct strain of Culex Flavivirus from mosquitoes collected in Mainland China.* Virol J, 2012. **9**: p. 73.
80. Huhtamo, E., et al., *Novel insect-specific flavivirus isolated from northern Europe.* Virology, 2012. **433**(2): p. 471-8.
81. Huhtamo, E., et al., *Novel flaviviruses from mosquitoes: mosquito-specific evolutionary lineages within the phylogenetic group of mosquito-borne flaviviruses.* Virology, 2014. **464-465**: p. 320-329.
82. Iwashita, H., et al., *Mosquito arbovirus survey in selected areas of Kenya: detection of insect-specific virus.* Trop Med Health, 2018. **46**: p. 19.
83. Jeffries, C.L., et al., *Detection of Cell-Fusing Agent virus across ecologically diverse populations of Aedes aegypti on the Caribbean island of Saint Lucia.* Wellcome Open Res, 2020. **5**: p. 149.
84. Kallies, R., et al., *Genetic characterization of goutanap virus, a novel virus related to negeviruses, cileviruses and higreviruses.* Viruses, 2014. **6**(11): p. 4346-57.

85. Kawakami, K., et al., *Characterization of a novel negevirus isolated from Aedes larvae collected in a subarctic region of Japan.* Arch Virol, 2016. **161**(4): p. 801-9.
86. Kenney, J.L., et al., *Characterization of a novel insect-specific flavivirus from Brazil: potential for inhibition of infection of arthropod cells with medically important flaviviruses.* J Gen Virol, 2014. **95**(Pt 12): p. 2796-2808.
87. Kim, D.Y., et al., *Characterization of Culex Flavivirus (Flaviviridae) strains isolated from mosquitoes in the United States and Trinidad.* Virology, 2009. **386**(1): p. 154-9.
88. Klein, A., et al., *Screening for Viruses and Lemur-Associated Filara in Wild-Caught Mosquitoes From Madagascar.* J Med Entomol, 2021. **58**(2): p. 983-989.
89. Kobayashi, D., et al., *Isolation and characterization of a new iflavirus from Armigeres spp. mosquitoes in the Philippines.* J Gen Virol, 2017. **98**(11): p. 2876-2881.
90. Kopp, A., et al., *Detection of Two Highly Diverse Peribunyaviruses in Mosquitoes from Palenque, Mexico.* Viruses, 2019. **11**(9).
91. Kubacki, J., et al., *Viral Metagenomic Analysis of Aedes albopictus Mosquitos from Southern Switzerland.* Viruses, 2020. **12**(9).
92. Kuwata, R., et al., *Characterization of Dak Nong virus, an insect nidovirus isolated from Culex mosquitoes in Vietnam.* Arch Virol, 2013. **158**(11): p. 2273-84.
93. Kuwata, R., et al., *Analysis of Mosquito-Borne Flavivirus Superinfection in Culex tritaeniorhynchus (Diptera: Culicidae) Cells Persistently Infected with Culex Flavivirus (Flaviviridae).* J Med Entomol, 2015. **52**(2): p. 222-9.
94. Kyaw Kyaw, A., et al., *Isolation and genomic characterization of Culex flaviviruses from mosquitoes in Myanmar.* Virus Res, 2018. **247**: p. 120-124.
95. Lara Pinto, A.Z., et al., *Novel viruses in salivary glands of mosquitoes from sylvatic Cerrado, Midwestern Brazil.* PLoS One, 2017. **12**(11): p. e0187429.
96. Liang, W., et al., *Distribution and phylogenetic analysis of Culex flavivirus in mosquitoes in China.* Arch Virol, 2015. **160**(9): p. 2259-68.
97. Liu, L., et al., *Comparative viromes of Culicoides and mosquitoes reveal their consistency and diversity in viral profiles.* Brief Bioinform, 2021. **22**(4).
98. Ma, M., et al., *Discovery of DNA viruses in wild-caught mosquitoes using small RNA high throughput sequencing.* PLoS One, 2011. **6**(9): p. e24758.
99. Machado, D.C., et al., *First identification of Culex flavivirus (Flaviviridae) in Brazil.* Intervirology, 2012. **55**(6): p. 475-83.
100. Maia, L.M.S., et al., *Novel Viruses in Mosquitoes from Brazilian Pantanal.* Viruses, 2019. **11**(10).
101. Marklewitz, M., et al., *Evolutionary and phenotypic analysis of live virus isolates suggests arthropod origin of a pathogenic RNA virus family.* Proc Natl Acad Sci U S A, 2015. **112**(24): p. 7536-41.
102. Martin, E., et al., *Mosquito-Borne Viruses and Insect-Specific Viruses Revealed in Field-Collected Mosquitoes by a Monitoring Tool Adapted from a Microbial Detection Array.* Appl Environ Microbiol, 2019. **85**(19).
103. Martin, E., et al., *Cell fusing agent virus (Flavivirus) infection in Aedes aegypti in Texas: seasonality, comparison by trap type, and individual viral loads.* Arch Virol, 2020. **165**(8): p. 1769-1776.
104. McLean, B.J., et al., *Differential suppression of persistent insect specific viruses in trans-infected wMel and wMelPop-CLA Aedes-derived mosquito lines.* Virology, 2019. **527**: p. 141-145.
105. Miranda, J., et al., *First report of Culex flavivirus infection from Culex coronator (Diptera: Culicidae), Colombia.* Virol J, 2019. **16**(1): p. 1.
106. Misencik, M.J., et al., *Isolation of a Novel Insect-Specific Flavivirus from Culiseta melanura in the Northeastern United States.* Vector Borne Zoonotic Dis, 2016. **16**(3): p. 181-90.
107. Moraes, O.S., et al., *Natural infection by Culex flavivirus in Culex quinquefasciatus mosquitoes captured in Cuiabá, Mato Grosso Mid-Western Brazil.* Med Vet Entomol, 2019. **33**(3): p. 397-406.

108. Morais, P., et al., *Insect-specific flaviviruses and densoviruses, suggested to have been transmitted vertically, found in mosquitoes collected in Angola: Genome detection and phylogenetic characterization of viral sequences.* Infect Genet Evol, 2020. **80**: p. 104191.
109. Munivenkatappa, A., et al., *Identification of Phasi Charoen-Like Phasivirus in Field Collected Aedes aegypti from Karnataka State, India.* Vector Borne Zoonotic Dis, 2021. **21**(11): p. 900-909.
110. Muttis, E., et al., *First record of a mosquito iridescent virus in Culex pipiens L. (Diptera: Culicidae).* Arch Virol, 2012. **157**(8): p. 1569-71.
111. Nabeshima, T., et al., *Tanay virus, a new species of virus isolated from mosquitoes in the Philippines.* J Gen Virol, 2014. **95**(Pt 6): p. 1390-1395.
112. Nebbak, A., et al., *Virome Diversity among Mosquito Populations in a Sub-Urban Region of Marseille, France.* Viruses, 2021. **13**(5).
113. Newton, N.D., et al., *Genetic, Morphological and Antigenic Relationships between Mesonivirus Isolates from Australian Mosquitoes and Evidence for Their Horizontal Transmission.* Viruses, 2020. **12**(10).
114. Ng, T.F., et al., *Broad surveys of DNA viral diversity obtained through viral metagenomics of mosquitoes.* PLoS One, 2011. **6**(6): p. e20579.
115. Obara-Nagoya, M., et al., *Ecological and genetic analyses of the complete genomes of Culex flavivirus strains isolated from Culex tritaeniorhynchus and Culex pipiens (Diptera: Culicidae) group mosquitoes.* J Med Entomol, 2013. **50**(2): p. 300-9.
116. O'Brien, C.A., et al., *Discovery and Characterisation of Castlerea Virus, a New Species of Negevirus Isolated in Australia.* Evol Bioinform Online, 2017. **13**: p. 1176934317691269.
117. O'Brien, C.A., et al., *A Unique Relative of Rotifer Birnavirus Isolated from Australian Mosquitoes.* Viruses, 2020. **12**(9).
118. Öhlund, P., et al., *Viromics Reveal a Number of Novel RNA Viruses in Swedish Mosquitoes.* Viruses, 2019. **11**(11).
119. Öncü, C., et al., *West Nile virus, Anopheles flavivirus, a novel flavivirus as well as Merida-like rhabdovirus Turkey in field-collected mosquitoes from Thrace and Anatolia.* Infect Genet Evol, 2018. **57**: p. 36-45.
120. Orba, Y., et al., *Diverse mosquito-specific flaviviruses in the Bolivian Amazon basin.* J Gen Virol, 2021. **102**(3).
121. Papa, A., et al., *Insect-specific flaviviruses in Aedes mosquitoes in Greece.* Arch Virol, 2016. **161**(8): p. 2183-8.
122. Parreira, R., et al., *Genetic characterization of an insect-specific flavivirus isolated from Culex theileri mosquitoes collected in southern Portugal.* Virus Res, 2012. **167**(2): p. 152-61.
123. Parry, R., et al., *Identification and RNAi Profile of a Novel Iflavirus Infecting Senegalese Aedes vexans arabiensis Mosquitoes.* Viruses, 2020. **12**(4).
124. Pettersson, J.H., et al., *Meta-Transcriptomic Comparison of the RNA Viromes of the Mosquito Vectors Culex pipiens and Culex torrentium in Northern Europe.* Viruses, 2019. **11**(11).
125. Pyke, A.T., et al., *Uncovering the genetic diversity within the Aedes notoscriptus virome and isolation of new viruses from this highly urbanised and invasive mosquito.* Virus Evol, 2021. **7**(2): p. veab082.
126. Ramírez, A.L., et al., *Metagenomic Analysis of the Virome of Mosquito Excreta.* mSphere, 2020. **5**(5).
127. Ramos-Nino, M.E., et al., *High prevalence of Phasi Charoen-like virus from wild-caught Aedes aegypti in Grenada, W.I. as revealed by metagenomic analysis.* PLoS One, 2020. **15**(1): p. e0227998.
128. Ramos-Nino, M.E., et al., *Metagenomic analysis of Aedes aegypti and Culex quinquefasciatus mosquitoes from Grenada, West Indies.* PLoS One, 2020. **15**(4): p. e0231047.

129. Reuter, G., et al., *Detection and genome analysis of a novel (dima)rhabdovirus (Riverside virus) from Ochlerotatus sp. mosquitoes in Central Europe.* Infect Genet Evol, 2016. **39**: p. 336-341.
130. Ribeiro, M.S., et al., *High Prevalence of a Newly Discovered Wutai Mosquito Phasivirus in Mosquitoes from Rio de Janeiro, Brazil.* Insects, 2019. **10**(5).
131. Ribeiro, G.O., et al., *Detection of RNA-Dependent RNA Polymerase of Hubei Reo-Like Virus 7 by Next-Generation Sequencing in Aedes aegypti and Culex quinquefasciatus Mosquitoes from Brazil.* Viruses, 2019. **11**(2).
132. Ribeiro, G.O., et al., *Aedes aegypti from Amazon Basin Harbor High Diversity of Novel Viral Species.* Viruses, 2020. **12**(8).
133. Rizzo, F., et al., *Molecular characterization of flaviviruses from field-collected mosquitoes in northwestern Italy, 2011-2012.* Parasit Vectors, 2014. **7**: p. 395.
134. Rwegoshora, R.T., K.J. Baisley, and P. Kittayapong, *Seasonal and spatial variation in natural densovirus infection in Anopheles minimus S.L. in Thailand.* Southeast Asian J Trop Med Public Health, 2000. **31**(1): p. 3-9.
135. Sadeghi, M., et al., *Virome of > 12 thousand Culex mosquitoes from throughout California.* Virology, 2018. **523**: p. 74-88.
136. Sadeghi, M., et al., *Genomes of viral isolates derived from different mosquitos species.* Virus Res, 2017. **242**: p. 49-57.
137. Sanborn, M.A., et al., *Metagenomic analysis reveals Culex mosquito virome diversity and Japanese encephalitis genotype V in the Republic of Korea.* Mol Ecol, 2021. **30**(21): p. 5470-5487.
138. Sanborn, M.A., et al., *Metagenomic Analysis Reveals Three Novel and Prevalent Mosquito Viruses from a Single Pool of Aedes vexans nipponii Collected in the Republic of Korea.* Viruses, 2019. **11**(3).
139. Scarpassa, V.M., et al., *An insight into the sialotranscriptome and virome of Amazonian anophelines.* BMC Genomics, 2019. **20**(1): p. 166.
140. Schuster, S., et al., *A unique nodavirus with novel features: mosinovirus expresses two subgenomic RNAs, a capsid gene of unknown origin, and a suppressor of the antiviral RNA interference pathway.* J Virol, 2014. **88**(22): p. 13447-59.
141. Shahhosseini, N., et al., *Detection and characterization of a novel rhabdovirus in Aedes cantans mosquitoes and evidence for a mosquito-associated new genus in the family Rhabdoviridae.* Infect Genet Evol, 2017. **55**: p. 260-268.
142. Shi, M., et al., *High-Resolution Metatranscriptomics Reveals the Ecological Dynamics of Mosquito-Associated RNA Viruses in Western Australia.* J Virol, 2017. **91**(17).
143. Shi, C., et al., *Stable distinct core eukaryotic viromes in different mosquito species from Guadeloupe, using single mosquito viral metagenomics.* Microbiome, 2019. **7**(1): p. 121.
144. Shi, C., et al., *Stability of the Virome in Lab- and Field-Collected Aedes albopictus Mosquitoes across Different Developmental Stages and Possible Core Viruses in the Publicly Available Virome Data of Aedes Mosquitoes.* mSystems, 2020. **5**(5).
145. Shi, C., et al., *A metagenomic survey of viral abundance and diversity in mosquitoes from Hubei province.* PLoS One, 2015. **10**(6): p. e0129845.
146. Silva, M., et al., *A diverse assemblage of RNA and DNA viruses found in mosquitoes collected in southern Portugal.* Virus Res, 2019. **274**: p. 197769.
147. Stanojević, M., et al., *Depicting the RNA Virome of Hematophagous Arthropods from Belgrade, Serbia.* Viruses, 2020. **12**(9).
148. Supriyono, et al., *Mosquito-borne viruses, insect-specific flaviviruses (family Flaviviridae, genus Flavivirus), Banna virus (family Reoviridae, genus Seadornavirus), Bogor virus (unassigned member of family Permutotetraviridae), and alphamesoniviruses 2 and 3 (family Mesoniviridae, genus Alphamesonivirus) isolated from Indonesian mosquitoes.* J Vet Med Sci, 2020. **82**(7): p. 1030-1041.
149. Suvanto, M.T., et al., *A novel negevirus isolated from Aedes vexans mosquitoes in Finland.* Arch Virol, 2020. **165**(12): p. 2989-2992.

150. Takhampunya, R., et al., *Distribution and mosquito hosts of Chaoyang virus, a newly reported flavivirus from the Republic of Korea, 2008-2011.* J Med Entomol, 2014. **51**(2): p. 464-74.
151. Tangudu, C.S., et al., *Skunk River virus, a novel orbivirus isolated from Aedes trivittatus in the United States.* J Gen Virol, 2019. **100**(2): p. 295-300.
152. Tesh, R.B., et al., *Characterization of Port Bolivar Virus, a Novel Entomobirnavirus (Birnaviridae) Isolated from Mosquitoes Collected in East Texas, USA.* Viruses, 2020. **12**(4).
153. Thannesberger, J., et al., *Highly Sensitive Virome Characterization of Aedes aegypti and Culex pipiens Complex from Central Europe and the Caribbean Reveals Potential for Interspecies Viral Transmission.* Pathogens, 2020. **9**(9).
154. Thongsripong, P., et al., *Metagenomic shotgun sequencing reveals host species as an important driver of virome composition in mosquitoes.* Sci Rep, 2021. **11**(1): p. 8448.
155. Tyler, S., et al., *Distribution and phylogenetic comparisons of a novel mosquito flavivirus sequence present in Culex tarsalis Mosquitoes from western Canada with viruses isolated in California and Colorado.* Am J Trop Med Hyg, 2011. **85**(1): p. 162-8.
156. Vasilakis, N., et al., *Mesoniviruses are mosquito-specific viruses with extensive geographic distribution and host range.* Virol J, 2014. **11**: p. 97.
157. Villinger, J., et al., *Arbovirus and insect-specific virus discovery in Kenya by novel six genera multiplex high-resolution melting analysis.* Mol Ecol Resour, 2017. **17**(3): p. 466-480.
158. Wang, Y., et al., *Complete genome sequence of a novel negevirus isolated from Culex tritaeniorhynchus in China.* Arch Virol, 2019. **164**(3): p. 907-911.
159. Wang, L., et al., *Establishment of Culex modestus in Belgium and a Glance into the Virome of Belgian Mosquito Species.* mSphere, 2021. **6**(2).
160. Warrilow, D., et al., *The taxonomy of an Australian nodavirus isolated from mosquitoes.* PLoS One, 2018. **13**(12): p. e0210029.
161. Wastika, C.E., et al., *Discoveries of Exoribonuclease-Resistant Structures of Insect-Specific Flaviviruses Isolated in Zambia.* Viruses, 2020. **12**(9).
162. Williams, S.H., et al., *Discovery of Jogalong virus, a novel hepacivirus identified in a Culex annulirostris (Skuse) mosquito from the Kimberley region of Western Australia.* PLoS One, 2020. **15**(1): p. e0227114.
163. Williams, S.H., et al., *The Diversity and Distribution of Viruses Associated with Culex annulirostris Mosquitoes from the Kimberley Region of Western Australia.* Viruses, 2020. **12**(7).
164. Xia, H., et al., *Comparative Metagenomic Profiling of Viromes Associated with Four Common Mosquito Species in China.* Virol Sin, 2018. **33**(1): p. 59-66.
165. Xiao, P., et al., *Metagenomic Sequencing From Mosquitoes in China Reveals a Variety of Insect and Human Viruses.* Front Cell Infect Microbiol, 2018. **8**: p. 364.
166. Yamao, T., et al., *Novel virus discovery in field-collected mosquito larvae using an improved system for rapid determination of viral RNA sequences (RDV ver4.0).* Arch Virol, 2009. **154**(1): p. 153-8.
167. Yezli, S., et al., *Lack of Zika Virus and Other Recognized Flaviviruses among the Mosquito Vectors during and Post the Hajj Mass Gathering.* Int J Environ Res Public Health, 2021. **18**(12).
168. Zakrzewski, M., et al., *Mapping the virome in wild-caught Aedes aegypti from Cairns and Bangkok.* Sci Rep, 2018. **8**(1): p. 4690.
169. Zana, B., et al., *Molecular traces of a putative novel insect flavivirus from Anopheles hyrcanus mosquito species in Hungary.* Acta Virol, 2017. **61**(1): p. 127-129.
170. Zhai, Y.G., et al., *Isolation and characterization of the full coding sequence of a novel densovirus from the mosquito Culex pipiens pallens.* J Gen Virol, 2008. **89**(Pt 1): p. 195-199.

171. Zhang, G., et al., *Cell fusing agent virus and dengue virus mutually interact in Aedes aegypti cell lines.* Sci Rep, 2017. **7**(1): p. 6935.
172. Zhang, X., et al., *Discovery and high prevalence of Phasi Charoen-like virus in field-captured Aedes aegypti in South China.* Virology, 2018. **523**: p. 35-40.
173. Zhao, L., et al., *Characterization of a Novel Tanay Virus Isolated From Anopheles sinensis Mosquitoes in Yunnan, China.* Front Microbiol, 2019. **10**: p. 1963.
174. Zhao, M., et al., *Viral metagenomics unveiled extensive communications of viruses within giant pandas and their associated organisms in the same ecosystem.* Sci Total Environ, 2022: p. 153317.
175. Zuo, S., et al., *Detection of Quang Binh virus from mosquitoes in China.* Virus Res, 2014. **180**: p. 31-8.
