# Supplementary material for: Composition and global distribution of the mosquito virome - A comprehensive database of insect-specific viruses
Source: One Health. 2023 Jan 20;16:100490. doi: 10.1016/j.onehlt.2023.100490 (PMC9929601; doi:10.1016/j.onehlt.2023.100490)
Supplement: Supplementary Table S2 — Virus positive mosquito species, with the corresponding number of continents, countries and studies in which they are sampled. [file mmc3.pdf]

Supplementary Table S2. Virus positive mosquito species, with the corresponding number of continents, countries and studies in which they are sampled.

Genus	Species	Continents	Countries	Studies
**Aedeomyia**	catasticta	1	1	1
**Aedes**	aegypti	5	17	31
	alboannulatus	1	1	3
	albopictus	4	10	19
	annulipes	2	2	2
	atlanticus	1	1	1
	bekkui	1	1	1
	berlandi	1	1	1
	caballus	1	1	1
	camptorhynchus	1	1	3
	cantans	1	2	2
	caspius	2	4	6
	cinereus	1	1	1
	clelandi	1	1	1
	communis	1	1	1
	cumminsii	1	1	1
	detritus	1	1	1
	dorsalis	2	2	2
	flavescens	1	1	1
	flavidorsalis	1	1	1
	flavopictus	1	1	1
	fowleri	1	1	1
	fucifer	1	1	1
	hesperonotius	1	1	2
	luteocephalus	1	1	1
	mcintoshi	1	1	1
	minutus	1	1	1
	normanensis	1	1	3
	notoscriptus	1	1	3
	procax	1	1	1
	scapularis	1	4	5
	sollicitans	1	1	1
	sudanensis	1	1	1
	taeniorhynchus	1	1	3
	terrens	1	1	1
	treplicates	1	1	1
	tricholabis	1	1	1
	triseriatus	1	1	1
	trivittatus	1	1	1
	turneri	1	1	1
	unknown	5	8	11
	vexans	4	7	9

	vigilax	1	1	4
	vittatus	1	1	1
**Anopheles**	amictus	1	1	1
	annulipes	1	1	2
	coluzzii	1	1	1
	coustani	1	2	2
	crucians	1	1	1
	cruzii	1	1	1
	darlingi	1	1	1
	funestus	1	1	1
	gambiae	1	4	5
	hyrcanus	1	1	1
	maculipalpis	1	1	1
	maculipennis	1	1	1
	marajoara	1	1	1
	minimus	1	2	2
	pharoensis	1	1	1
	pretoriensis	1	1	1
	rufipes	1	1	1
	sinensis	1	1	9
	squamosus	1	2	2
	stephensi	1	1	1
	tessellatus	1	1	1
	triannulatus	1	1	2
	unknown	4	9	8
	vagus	1	1	1
**Armigeres**	obturbans	1	1	1
	subalbatus	1	4	5
	unknown	1	1	2
**Coquillettidia**	fuscopennata	1	1	1
	juxtamansonia	1	1	1
	metallica	1	2	2
	richiardii	1	1	1
	xanthogaster	1	1	3
**Culex**	annulirostris	2	2	11
	antennatus	1	3	3
	australicus	1	1	3
	bitaeniorhynchus	1	1	1
	chidesteri	1	1	1
	coronator	1	3	3
	decens	1	2	2
	declarator	2	3	3
	erythrothorax	1	1	2
	fatigans	1	1	1
	fuscocephala	1	3	4

	globocoxitus	1	1	3
	inatomii	1	2	2
	laticinctus	1	1	1
	modestus	2	2	2
	neavei	1	1	1
	nebulosus	1	3	4
	nigripalpus	2	3	3
	orientalis	1	1	1
	pipiens	5	18	34
	poicilipes	1	1	1
	pseudovishnui	1	2	2
	pullus	1	1	2
	quinquefasciatus	5	16	29
	restuans	1	1	1
	sitiens	2	2	4
	tarsalis	1	2	5
	theileri	1	2	3
	torrentium	1	1	2
	tritaeniorhynchus	2	8	23
	univittatus	1	3	3
	unknown	6	16	30
	vagans	1	2	2
	vishnui	1	3	4
**Culiseta**	annulata	1	2	2
	atra	1	1	1
	incidens	1	1	1
	longiareolata	1	2	2
	melanura	1	1	1
	unknown	1	1	1
**Heamagogus**	janthinomys	1	1	1
**Mansonia**	africana	1	4	4
	titillans	1	1	1
	uniformis	3	6	6
	unknown	3	3	4
	wilsoni	1	1	2
**Ochlerotatus**	fulvus	1	1	1
	koreicus	1	1	1
	sierrensis	1	1	1
	unknown	1	2	2
**Psorophora**	albigenu	1	2	2
	albipes	1	1	1
	ciliate	1	1	1
	columbiae	1	1	1
	ferox	1	1	1
	unknown	1	1	1

**Sabethes**	belisarioi	1	1	1
	gymonothorax	1	1	1
**Unknown**	unknown	6	15	24
**Uranotaenia**	unguiculata	1	1	2
	unknown	1	1	1
**Wyeomyia**	complosa	1	1	1
