# Supplementary figures and images for: Composition and global distribution of the mosquito virome - A comprehensive database of insect-specific viruses
Source: One Health. 2023 Jan 20;16:100490. doi: 10.1016/j.onehlt.2023.100490 (PMC9929601; doi:10.1016/j.onehlt.2023.100490)

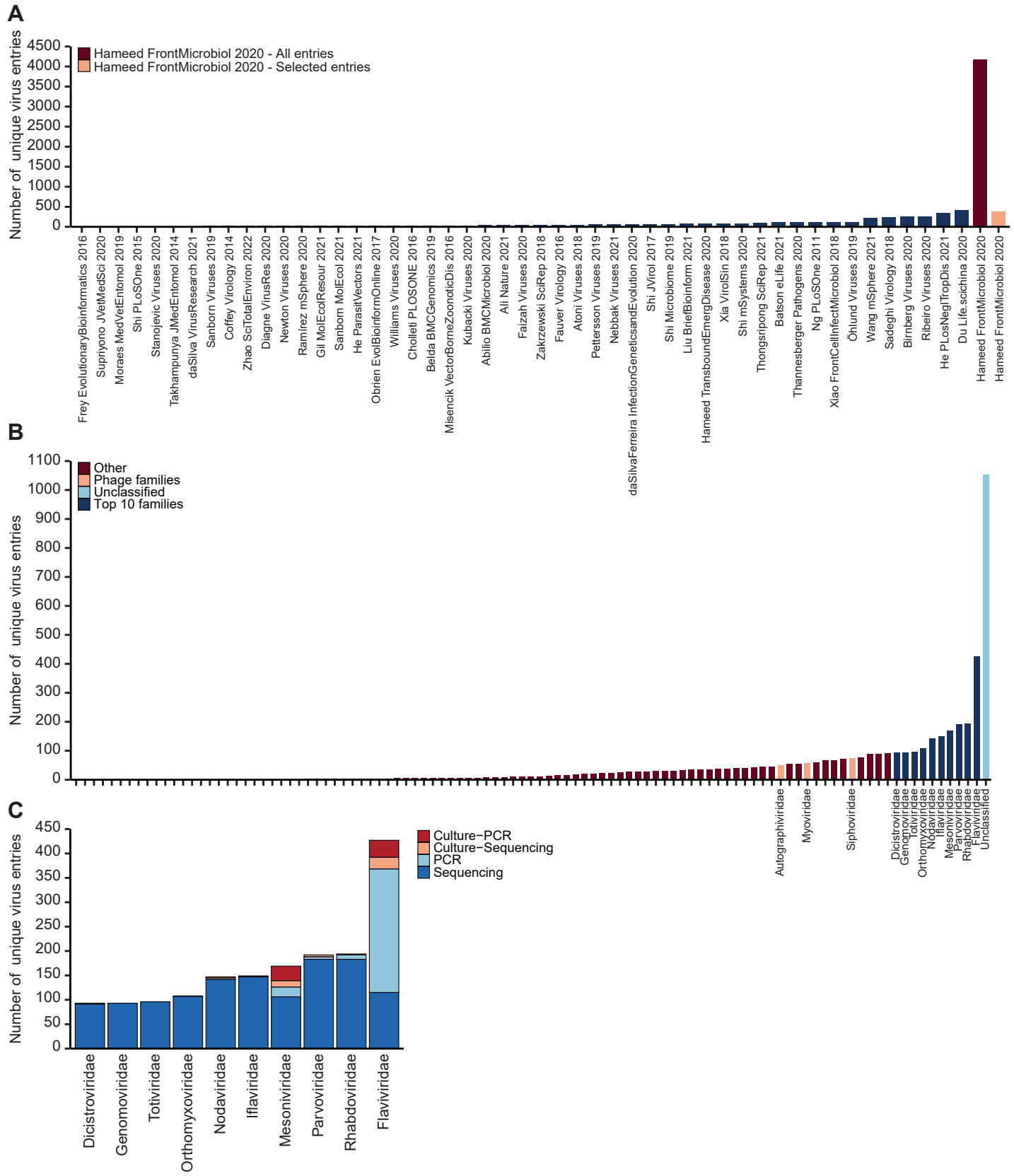

Supplement: Supplementary Fig. S1 — Unique virus entries in mosquito virome studies. (A) Number of unique virus entries reported for the top 50 studies that contributed most entries in our database. Unique virus entries for Hameed et al. (2020) before (dark red) and after (salmon) data curation are indicated. (B) Number of unique virus entries corresponding to the individual virus families for all mosquito species in our database. Bacteriophage families are indicated with salmon fill color; viruses not classified at the family level in light blue. (C) Number of unique virus entries for the top 10 most frequently detected families, according to detection method. [file mmc4.pdf]
